# Inferring probabilistic miRNA–mRNA interaction signatures in cancers: a role-switch approach

**DOI:** 10.1093/nar/gku182

**Published:** 2014-03-07

**Authors:** Yue Li, Cheng Liang, Ka-Chun Wong, Ke Jin, Zhaolei Zhang

**Affiliations:** 1 Department of Computer Science, University of Toronto, Toronto, Ontario M5S 3G4, Canada; 2 The Donnelly Centre, University of Toronto, Toronto, ON M5S 3E1, Canada; 3 Department of Molecular Genetics, University of Toronto, Toronto, ON M5S 1A4, Canada; 4 Banting and Best Department of Medical Research, University of Toronto, Toronto, ON M5S 3E1, Canada

## Abstract

Aberrant microRNA (miRNA) expression is implicated in tumorigenesis. The underlying mechanisms are unclear because the regulations of each miRNA on potentially hundreds of mRNAs are sample specific. We describe a novel approach to infer *Pro*babilistic *Mi*RNA–mRNA *I*nteraction *S*ignatur*e* (‘ProMISe’) from a single pair of miRNA–mRNA expression profile. Our model considers mRNA and miRNA competition as a probabilistic function of the expressed seeds (matches). To demonstrate ProMISe, we extensively exploited The Cancer Genome Atlas data. As a target predictor, ProMISe identifies more confidence/validated targets than other methods. Importantly, ProMISe confers higher cancer diagnostic power than using expression profiles alone. Gene set enrichment analysis on averaged ProMISe uniquely revealed respective target enrichments of oncomirs miR-21 and 145 in glioblastoma and ovarian cancers. Moreover, comparing matched breast (BRCA) and thyroid (THCA) tumor/normal samples uncovered thousands of tumor-related interactions. For example, ProMISe–BRCA network involves miR-155/183/21, which exhibits higher ProMISe coupled with coherently higher miRNA expression and lower target expression; oncomirs miR-221/222 in the ProMISe–THCA network engage with many downregulated target genes. Together, our probabilistic approach of integrating expression and sequence scores establishes a functional link between the aberrant miRNA and mRNA expression, which was previously under-appreciated due to the methodological differences.

## INTRODUCTION

MicroRNAs (miRNAs) are small (∼22 nucleotides) RNA molecules that base-pair with mRNA primarily at the 3′ untranslated region (UTR) to cause mRNA degradation or translational repression (1). Recent studies have linked alterations in miRNA expression with various cancers (2–3). Functional characterization of miRNAs depends on precise identification of their targets. Earlier developed miRNA target prediction programs are mostly based on sequence complementarity, evolutionary conservation, free energy and/or target site accessibility (4). Although useful, these sequence-based methods often suffer from high false positive rate and are unable to capture sample-specific interactions. More recently developed methods have incorporated mRNA and miRNA expression data generated by microarrays or RNA-seq to predict functional miRNA–mRNA interactions (MMIs). Despite diverse modeling approaches, a majority of the expression-based methods rely on negative expression correlation between miRNA and mRNA. In terms of model complexity, these methods range from the simplest Pearson correlation to more sophisticated Bayesian method. In particular, GenMiR++ is based on variational Bayesian to infer the posterior probabilities of MMIs as represented by the linear coefficients in a regression framework (5). Regularized least-squares linear regression such as LASSO has also been used to calculate a sparse linear solution of the most significant MMI (6).

While a step forward from the sequence-based methods, there are two important limitations in the current expression-based methods. First, these methods usually require a large number of samples to compute MMIs. Thus, they have difficulty in identifying ‘personalized’ MMIs in individual samples. Indeed, each tissue or cell line has a unique miRNA regulatory network with weighted MMI edges, which can be used as molecular signatures similar to the uniqueness of mRNA/miRNA expression profile (2,7). Second, while most methods take into account the potential competition among miRNAs for the same mRNA in regression models, the reciprocal competition among mRNAs for the same miRNA has not been systematically addressed. Yet both competitions are experimentally supported. For the former, not only the endogenous miRNAs may compete for the same mRNA harboring overlapping seed matches but also for the limited Argonaute (Ago), the catalytic component of the RNA silencing complex (RISC) (8). For the latter competition, Arvey *et al.* (9) showed that miRNAs that have a higher number of available target transcripts will downregulate each individual target gene to a lesser extent than those with a lower number of targets. In other words, the affected mRNA target population ‘dilutes’ the individual miRNA effect by sharing target sites among them.

**Figure 1.**
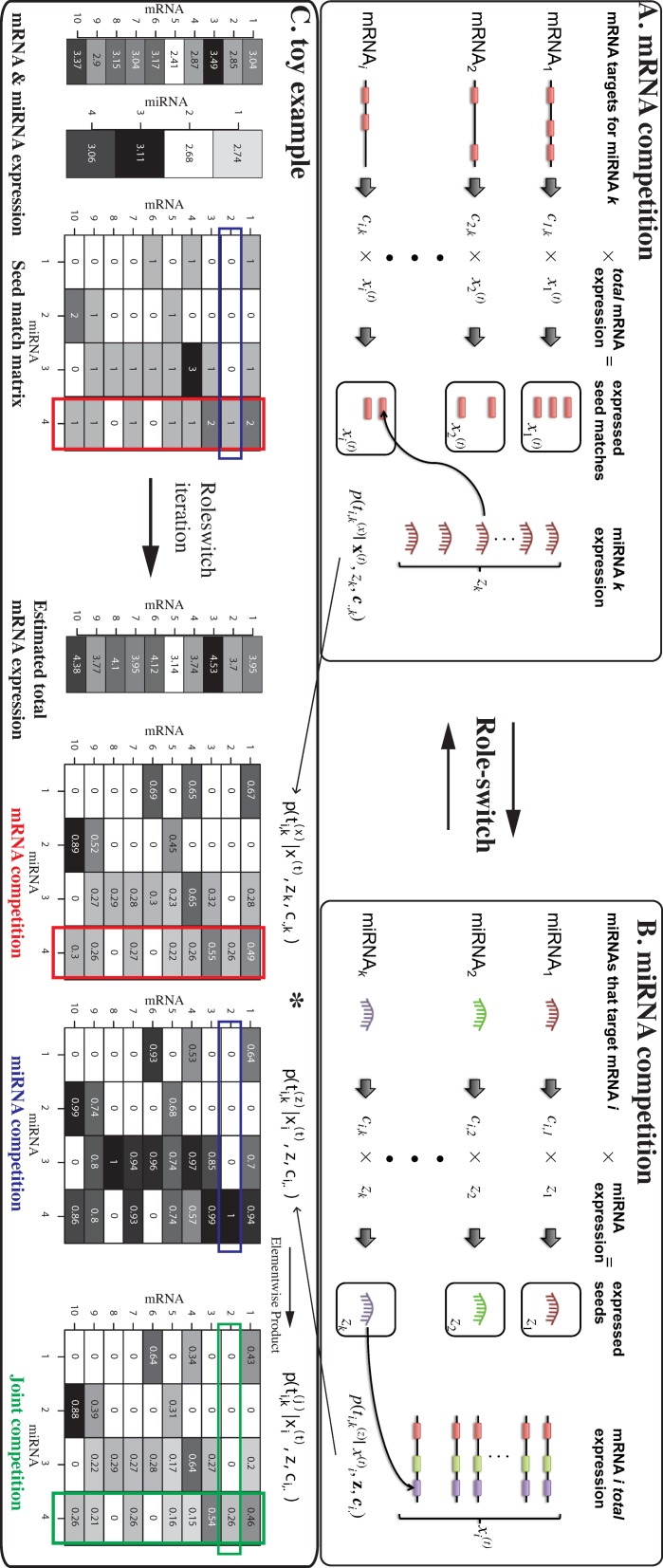
The proposed competition model schema. (**A**) Expressed seed match of mRNA *i* for miRNA *k* is defined as the product of the number of target sites *c*_*i*, *k*_ and the total expression of mRNA *i*. The probability of mRNA *i* ‘attracting’ miRNA *k* takes into account the expression of miRNA *k* and the *total* expression of other mRNA ${\mathbf {x}}^{(t)}$ that carries compatible seed match for miRNA *k.* (**B**) Conversely, the expressed seed *k* for mRNA *i* is the number of target sites that miRNA *k* can recognize on mRNA *i* multiplied by the expression of miRNA *k*. The probability of miRNA *k* targeting mRNA *i* considers the ‘total’ expression of mRNA *i* and all of the other miRNA expression $\mathbf {z}$ that can recognize the target sites of mRNA *i*. (**C**) A toy example. The inputs are 10 simulated mRNAs and 4 miRNA expression and a 10 × 4 seed-match matrix. ProMISe estimates the total mRNA expression based on the mRNA competition. It also infers the miRNA competition based on the total mRNA and the observed miRNA expression. The joint competition is the element-wise product of the above two matrices. Colored rectangles highlight some model properties explained in the main text.

In this paper, we describe three related models via a novel approach inspired by a ‘role-switch’ analogy. The first (and second) model, namely ‘mRNA competition’ (and ‘miRNA competition’), takes into account the competitions among mRNAs (and miRNAs) for the same miRNA (and mRNA) using paired expression profile coupled with target site information (Figure 1). The third model ‘joint competition’ combines the former two predictions as joint probabilities. Using the expression data from (10) and The Cancer Genome Atlas (TCGA) (11), we first assess the proposed models as a target prediction tool by benchmarking the confidence or validated targets. The proposed models and the resulting probabilistic scores collectively termed as the *Pro*babilistic *Mi*RNA–mRNA *I*nteraction *S*ignatur*e* (ProMISe) confer competitive performance comparing with existing sequence- and regression-based methods. Furthermore, ProMISe signature exhibits competitive diagnostic power in discriminating normal/tumor profiles compared with using expression profiles alone. One explanation for the above observations is that ProMISe can capture complex MMIs not easily identified by examining expression profiles alone. For instance, some specific MMI changes in tumor are not only due to the expression changes of the corresponding miRNA/mRNA members but also due to the expression changes of the competing miRNAs and/or mRNAs. Moreover, genes with aberrant ProMISe signature are enriched for cancer-specific and oncomir-regulated gene sets based on gene set enrichment analysis (GSEA) (12). Some of the oncogenes with aberrant interactions do not exhibit significant expression changes. Thus, the inferred ProMISe signature can provide complementary information to the expression profiles. Integrative differential analysis of expression and ProMISe signature using matched tumor/normal samples from breast and thyroid cancers revealed many tumor-specific MMIs, involving canonical oncogenes and oncomirs. Together, our integrative approach bridges the aberrant miRNA and gene expression profiles with miRNA targeting mechanism, which enables us to explore cancer biology from a unique molecular perspective.

## MATERIALS AND METHODS

### ProMISe

We propose a novel probabilistic approach to infer probabilistic miRNA–mRNA interaction signature (ProMISe) using a ‘single’ pair of miRNA–mRNA expression profile. Assume we have *N* mRNAs and *M* miRNA. Let $\mathbf{x}$ and $\mathbf{z}$ be the observed mRNA and miRNA expression vectors, respectively, and $\mathbf{C}$ be the *N* × *M* seed-match matrix, where *c*_*i*, *k*_ denotes the number of target sites on mRNA *i* for miRNA *k*. Assuming the cell is at a condition-specific equilibrium state, where mRNA are being dynamically transcribed and degraded (partly due to miRNA binding), then the total amount of transcribed mRNA targets $\mathbf{x}^{(t)}$ are unobserved and higher than the observed (equilibrium) expression level $\mathbf{x}^{(o)}$. Our goal is to infer $p(t_{i,k} | \mathbf {x}^{(t)}, \mathbf {z}^{(t)}, \mathbf{C})$ for whether miRNA *k* targets mRNA *i*, given the (hidden) total transcription levels of mRNA and miRNA. Notably, $\mathbf {z}^{(t)} = \mathbf {z}^{(o)} \equiv \mathbf{z}$, assuming that the observed miRNA expression is the same as its total transcribed levels. Thus, we drop the superscript for $\mathbf{z}$ in the following formulation. Here we estimate $p(t_{i,k} | \mathbf {x}^{(t)}, \mathbf {z}^{(t)}, \mathbf{C})$ by three related models reflecting mRNA competition, miRNA competition, and joint competition, respectively. Let $\mathbf{c}_{.,k}$ and $\mathbf{c}_{i,.}$ denote the number of target sites of each mRNA for miRNA *k* and the number of target sites that mRNA *i* has for each miRNA, respectively. In the mRNA competition model, we express $p(t^{(x)}_{i,k} | \mathbf {x}^{(t)}, z_k, \mathbf{c}_{.,k})$ as the probability of mRNA *i* ‘attracting’ miRNA *k*, given the expression of all of the mRNAs that miRNA *k* can target according to $\mathbf{c}_{.,k}$ (Figure 1A). Thus, it reflects the *mRNA competition* for the same miRNA. Specifically, the probability that mRNA *i* attracting a specific miRNA *k* is calculated as the reversed probability that miRNA *k* is attracted by other mRNA *j* (*j* ≠ *i*):

(1) \begin{equation*}
p(t^{(x)}_{i,k} | {\bf x}^{(t)}, z_k, {\bf c}_{.,k}) = 1 - \left[\frac{\sum _{j\ne i}c_{j,k}x_j^{(t)}}{\sum _{j^{\prime }} c_{j^{\prime },k}x_{j^{\prime }}^{(t)}}\right]^{z_k}.
\end{equation*}

Note the use of the exponent $ z$_*k*_ from Equation (1) to reflect that the higher the miRNA *k* expressed the more likely it will be attracted to mRNA *i*. Conversely, the miRNA competition model $p(t^{(z)}_{i,k} | x_i^{(t)}, {\bf z}, \mathbf{c}_{i,.})$ reflects the probability that miRNA *k* targets mRNA *i*, taking into account the expression of all of the miRNAs that can target mRNA *i* according to $\mathbf{c}_{i,.}$ (Figure 1B). The probability that miRNA *k* targeting a particular mRNA *i* is calculated as the reversed probability that mRNA *i* is targeted by other miRNA *l* (*l* ≠ *k*):

(2) \begin{equation*} p(t^{(z)}_{i,k} | x_i^{(t)}, {\bf z}, {\bf c}_{i,.}) = 1 - \left[\frac{\sum _{l\ne k}c_{i,l}z_l}{\sum _{l^{\prime }} c_{i,l^{\prime }}z_{l^{\prime }}}\right]^{x_i^{(t)}}.
\end{equation*}

Thus, the first and second models respectively reflect the ‘dilution-effects’ of multiple mRNAs targeted by the same miRNA or the mRNA competition (9) and the competition among miRNAs for the same mRNA (13). Finally, we express the joint competition as $p(t_{i,k}^{(j)} | \mathbf {x}^{(t)}, \mathbf {z}, \mathbf{C})$:

(3) \begin{equation*} p(t_{i,k}^{(j)} | \mathbf {x}^{(t)}, \mathbf {z}, \mathbf {C}) = p(t^{(x)}_{i,k} | \mathbf {x}^{(t)}, z_k, \mathbf {c}_{.,k}) p(t^{(z)}_{i,k} | x_i^{(t)}, \mathbf {z}, \mathbf {c}_{i,.}).
\end{equation*}

Notably, an underlying assumption of Equation (3) is that mRNA competition and miRNA completion are independent events. Algorithmically, we estimate Equations (1–3) together in two phases. As initialization, we set $\mathbf {x}^{(t)} = \mathbf{x}^{(o)}$. We first estimate Equation (1). Given Equation (1), the expected reduction level due to miRNA *k* binding is estimated as

(4) \begin{equation*} \Delta x_{i,k} = \eta p(t^{(x)}_{i,k} | \mathbf {x}^{(t)}, z_k, \mathbf {c}_{.,k}) x_i^{(t)},
\end{equation*}

where η is the ‘learning rate’ (default: 0.001). Given Δ*x*_*i*, *k*_, the total transcribed mRNA is updated in two steps as follows:

(5) \begin{equation*} x_i^{(t)*} = x_i^{(t)} + \sum _k\Delta x_{i,k}
\end{equation*}

(6) \begin{equation*} x_i^{(t)} = \frac{x_i^{(t)*}}{\sum _ix_i^{(t)*}}T,
\end{equation*}

where Equation (5) reflects the total reduction of mRNA *i* by each miRNA and *T* defines the transcriptional capacity of the cell (default: $T = 1.3 \sum x_i^{(o)}$, where 1.3 is an arbitrary value that reflects the total transcribed mRNA ‘before’ miRNA repression). We then estimate Equation (2). The model alternates between estimating (1) and (2) until $p(t^{(x)}_{i,k} | \mathbf {x}^{(t)}, z_k, \mathbf{c}_{.,k})$ and $p(t^{(z)}_{i,k} | x_i^{(t)}, \mathbf {z}, \mathbf{c}_{i,.})$ increase by less than a threshold (tol) (default: 10^−5^) at *t*^th^ iteration:

\begin{eqnarray*} \max \left[\left| p(t^{(x)}_{i,k} | \mathbf {x}^{(t)}, z_k, \mathbf {c}_{.,k})^t - p(t^{(x)}_{i,k} | \mathbf {x}^{(t)}, z_k, \mathbf {c}_{.,k})^{t-1} \right|\right] \le {\rm tol} \\ \max \left[\left| p(t^{(z)}_{i,k} | x_i^{(t)}, \mathbf {z}, \mathbf {c}_{i,.})^t - p(t^{(z)}_{i,k} | x_i^{(t)}, \mathbf {z}, \mathbf {c}_{i,.})^{t-1} \right|\right] \le {\rm tol}. \end{eqnarray*}

### Target site information

We downloaded human target site information from TargetScanHuman 6.2 database (14). For each mRNA–miRNA pair, we calculated the number of corresponding conserved target sites. For multiple transcripts of the same gene, we used transcripts with the longest 3′UTR. The end result is an *N* × *M* seed-match matrix of *N* distinct mRNAs each corresponding to a distinct gene and *M* distinct miRNAs. We also obtained the context+ scores (CS) (15) and probability of conserved targeting (PCT) (14) as sequence-based scores for comparison.

### HEK293 test set and power analysis

Gene and miRNA expression from HEK293 as measured by serial analysis of gene expression (SAGE) and small RNA-seq were obtained from (10). We constructed the positive and negative target sets from the PAR-CLIP and microarray data generated by (10) following similar way described in (16). We first downloaded from doRiNAdb (17) the confidence AGO2 targets identified from PAR-CLIP data in the same study. We then downloaded from Gene Expression Omnibus (GEO) (GSE21577) the microarray data measuring gene expression in HEK293 after treated with mock control or a cocktail chemistry inhibiting 27 most highly expressed miRNA in HEK293. The true targets of the 27 miRNA are expected to exhibit increased expression level upon miRNA inhibition. Thus, the confidence positive targets were defined as the genes that are AGO2 targets, have at least one seed match to the 27 miRNA, and exhibit fold-change greater than 0.1. To create a confidence set of non-targets, we selected the same number of genes that are not AGO2 targets and exhibit non-positive fold-changes with priority given to genes with decreased expression. We then assessed the accuracy of the methods using receiver operating characteristic (ROC) and precision-recall curves (PRCs) (18) (details described in the simulation tests in Supplementary Data).

### TCGA data collection and processing

Expression data were downloaded from TCGA Data Portal (https://tcga-data.nci.nih.gov). Only the processed data (level 3) were used. To date, there are 10 cancer types that are associated with data both unrestricted for publication and containing paired miRNA and mRNA expression (Table 1). Except for glioblastoma multiforme (GBM) and ovarian serous cystadenocarcinoma (OV), for which the microarray data were used, RNA-seq(V2) and miRNA-seq were used for mRNA and miRNA expression data, respectively. Normal/tumor information for each sample were obtained from Biospecimen Metadata Browser (https://tcga-data.nci.nih.gov/uuid/uuidBrowser.htm) and mapped based on the sample ID. For the sequencing data, we used the RPKM (read per kilobase of exon per million mapped reads) values for mRNA and RPM (reads per million miRNA mapped) for miRNA. To ensure individual samples are comparable, we further quantile normalized the RPKM and RPM values within each disease type.

**Table 1.** Paired expression data from TCGA

Cancer	mRNA	miRNA	Normal	Tumor	Total
BRCA	13306	710	14	317	331
COAD	13306	710	0	177	177
GBM	10344	338	10	496	506
HNSC	13306	710	0	37	37
KIRC	13306	710	0	274	274
LUSC	13306	710	0	132	132
OV	8371	542	8	565	573
READ	13306	710	0	66	66
THCA	13306	710	58	485	543
UCEC	13306	710	1	124	125

BRCA: breast cancer; COAD: colon adenocarcinoma; GBM: glioblastoma multiforme; HNSC: head and neck squamous cell carcinoma; KIRC: clear cell carcinoma; LUSC: lung squamous cell carcinoma; OV: ovarian serous cystadenocarcinoma; READ: rectal adenocarcinoma; THCA: thyroid carcinoma; UCEC: uterine corpus endometrial carcinoma.

### Validated miRNA targets, oncogenes, oncomirs, OMIM genes and cancer gene hubs

Validated targets were downloaded from MirTarBase 3.5 (19), the oncogenes from COSMIC (20), the oncomirs from (3) and OMIM (Online Mendelian Inheritance in Man) genes from http://www.omim.org/. Putative cancer gene hubs, which are discriminative of luminal and basal subtypes of 16 breast cancer cell lines, were obtained from (21).

### Methods of comparison

**Figure 2.**
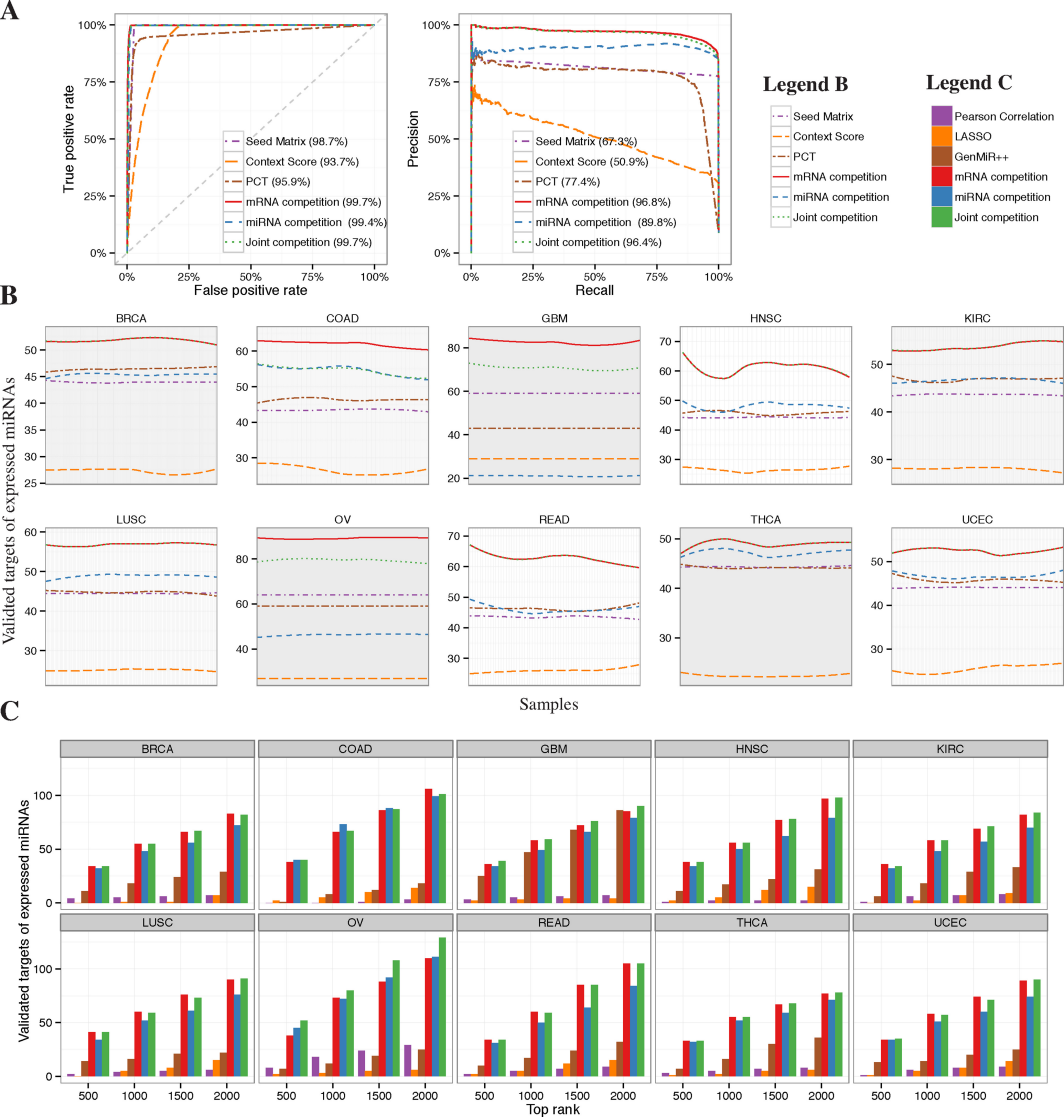
Identification of confidence or validated targets. (**A**) Based on positive/negative targets and the prediction scores, ROC and PRC were generated for the six methods, where Seed Matrix, TargetScan Context Score and PCT are sequence-based and the other three are proposed competition models. (**B**) The number of validated targets selected by each method among their top 1000 rankings as a function of sample. Ten panels correspond to 10 cancer types. The curves are smoothed using loess from R function geom_smooth. (**C**) Comparison of the competition models with other expression-based methods including Pearson correlation, LASSO and GenMiR++.

We compared ProMISe constructed from the three proposed competition models with six methods, namely Seed Matrix containing conserved target sites from TargetScan, TargetScan PCT, TargetScan Context Score, Pearson correlation, LASSO and GenMiR++. For Seed Matrix and TargetScan PCT/context score, which do not consider expression data, the mRNA–miRNA interactions were simply ranked by the corresponding number of target sites and specific scores. To be fair, we filtered out interactions involving non-expressed miRNA beforehand. Pearson correlation was computed using R built-in function cor. Here targets with negative correlation were ranked at the top. We implemented LASSO using *glmnet* with default parameters (i.e. α = 1 for LASSO) except that the best λ was chosen using cross-validation function cv.glmnet (22). The predictors in the LASSO model are the miRNA expression multiplied by the seed-match matrix. Thus, the expression of a miRNA has no effect on mRNA expression if the corresponding seed-matrix entry is zero. Here again targets with negative linear coefficients are ranked at the top. To run GenMiR++ (5) in Matlab, we converted the above seed matrix to binary matrix by setting nonzero target site count to 1. Due to the lack of validated targets, conventional ROC and PRC approaches cannot distinguish the method performances on TCGA data. Instead, we assessed each method by the number of validated targets in their top ranked 500–2000 targets with 500-interval (Figure 2).

### Unsupervised learning on cancer data

**Figure 3.**
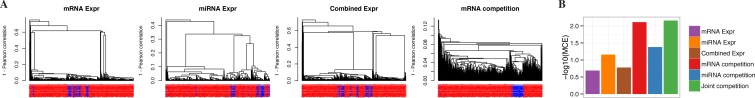
Cancer diagnosis. (**A**) Hierarchical clustering of expression or ProMISe signature for thyroid cancer. Red and blue colors indicate tumor and normal samples, respectively. (**B**) Regularized logistic regression was applied to classify normal and thyroid cancer tumor profiles using expression or ProMISe signature. Mean cross entropy (MCE) from LOOCV was used to assess the performance of each method. Superior method confers lower MCE and thus higher −log10(MCE).

Hierarchical clustering was applied to expression profiles or ProMISe signature using R function hclust (Figure 3A, Supplementary Figure S5). One minus Pearson correlation and average linkage were used.

### Supervised learning on cancer data

To classify samples into normal and cancer, we employed regularized logistic regression with L1/L2-norm using R package *glmnet* (22). Specifically, we trained a linear model with α = 0.5 on training set using gene expression, miRNA expression, combined expression or ProMISe signature from the three competition models. In each training process, the only free parameter λ was determined by 10-fold cross-validation using cv.glmnet. The performances of the trained models on the training and testing set were rigorously evaluated by leave-one-out cross-validation (LOOCV) in terms of mean cross entropy (MCE) error:

(7) \begin{equation*} {\rm MCE} = \frac{1}{N}\sum _{n=1}^N -t_n \log p_n
\end{equation*}

where *t* is a binary indicator for tumor (1) or normal (0) and *p* is the predictive probability from the logistic regression model for sample *n* of being tumor. Thus, MCE is small if both *t*_*n*_ and *p*_*n*_ are high or low at the same time and large when *t*_*n*_ is high and *p*_*n*_ is small. We applied LOOCV to THCA, which has at least 10% normal samples (Table 1). Figure 3B displays the resulting MCE in barplots.

### Permutation tests

The normal and tumor samples in THCA are not balanced (58 normals versus 485 tumors), which may lead to overestimation of model performance in terms of the MCE (Equation (7)). To assess the classifiers’ performances more rigorously, we conducted 100 permutation tests by randomly swapping values between normal and tumor samples and repeating the above LOOCV for each type of biomarkers. The empirical *p*-value of the permutation test is defined as the fraction of tests having MCE as least as good as the MCE derived from the real test.

### Gene set enrichment analysis

We applied GSEA (2.0.13) to both gene expression and genes with averaged ProMISe signature across miRNAs (12). Default setting was used. Gene sets were downloaded from the Molecular Signatures Database (MSigDB) (http://www.broadinstitute.org/gsea/msigdb/index.jsp). The maximum and minimum number of genes allowed in each gene set was set to 5000 and 15, respectively.

### Paired sample comparison

Paired *t*-test (*t*.test(…, paired = *T*) in R) was performed to compare quantile normalized ProMISe or expression profiles from the 14 and 58 matched tumor/normal samples in BRCA and THCA, respectively.

### Software availability

ProMISe was implemented as an R package available at Bioconductor: www.bioconductor.org/packages/release/bioc/html/Roleswitch.html.

## RESULTS

### ProMISe

The proposed model takes as inputs the paired mRNA and miRNA expression and the seed-match matrix. It then estimates the probabilities of different mRNA ‘attracting’ the same miRNA (i.e. mRNA competition; Figure 1A, Equation (1)), the probabilities of different miRNA targeting the same mRNA (i.e. miRNA competition; Figure 1B, Equation (2)), and the element-wise products of the above two (i.e. joint competition; Equation (3)). To highlight several important features of the proposed model, Figure 1C illustrates a toy example using simulated data of 10 mRNAs and 4 miRNAs. First, mRNA *i* that does not carry a seed match for miRNA *k* has zero probability of being its target (e.g. mRNA 1 and miRNA 2) (see Discussion section for other possibilities). Second, $p(t^{(x)}_{i,k} | \mathbf {x}^{(t)}, z_k, \mathbf{c}_{.,k})$ (mRNA competition) and $p(t^{(z)}_{i,k} | x_i^{(t)}, \mathbf {z}, \mathbf{c}_{i,.})$ (miRNA competition) differ for the same pair of miRNA and mRNA. For instance, $p(t^{(x)}_{2,4} | \mathbf {x}^{(t)}, z_4, \mathbf{c}_{.,4}) = 0.26$ and $p(t^{(z)}_{2,4} | x_1^{(t)}, \mathbf {z}, \mathbf{c}_{2,.}) = 1$. Intuitively, mRNA 2 has only one target site for miRNA 4, which can potentially target many other mRNAs (vertical red boxes) such as mRNA 1 and 3, that each has higher expression level, two target sites, and thus higher probabilities of being targeted by miRNA 4. On the other hand, mRNA 2 can only ‘attract’ miRNA 4 because none of the other three miRNAs recognizes its target site (horizontal blue boxes), which explains $p(t^{(z)}_{2,4} | x_2^{(t)}, \mathbf {z}, \mathbf{c}_{2,.}) = 1$. Third, the joint competition model (Equation (3)) provides a conservative estimate, for which both miRNA and mRNA competition scores must be high to confer a high confidence prediction (e.g. mRNA 10 and miRNA 2). The model converges quickly in only a few iterations for large number of mRNAs and miRNAs (Supplementary Figure S1). To rigorously test the models, we designed four scenarios (Supplementary Figure S2). Below we compared ProMISe with other methods using real expression data.

### Target predictions

We first compared ProMISe with sequence-based methods in discriminating the 1255 confidence positive and negative targets identified from HEK293 using the published data by (10) (Materials and Methods section). We chose TargetScan Context Score (15) and Probabilities of Conserved Targeting (PCT) (14) as the representative sequence-based methods based on our recent evaluations (4). We observed excellent Area Under the ROC Curve (AUROC) from all three proposed competition models (Figure 2A, left). In particular, the joint and mRNA competition models confer the highest AUROC (99.7%), which is 1–6% higher than the sequence-based methods. Additionally, we observed even better performance from the competition models in terms of precision recall (Figure 2A, right) with 19.4–45.9% improvements over the sequence-based methods. Interestingly, the mRNA competition model outperforms the joint and miRNA competition models by 0.4% and 7%, respectively. We next evaluated each model on the TCGA data. To further take advantage of the sequence information, we multiplied ProMISe with the TargetScan PCT. To be fair with the sequence-based methods, we filtered out interactions involving miRNAs that are not expressed in each sample beforehand. Each panel in Figure 2B depicts the number of validated targets among the top 1000 ranking as a function of sample ID. The ProMISe constructed from the mRNA competition model demonstrated clearly superior performances over other methods in identifying validated MMIs (Figure 2B). The joint competition model achieves similar performances as the mRNA competition model. Moreover, we observed consistent results using the top rankings from 500 to 2000 with 500-increment (Supplementary Figure S3). We then compared ProMISe with three other expression-based methods, namely Pearson correlation, LASSO regression (6) and GenMiR++ (5). Because ProMISe confers good pairwise correlation between individual samples (Supplementary Figure S4), the averaged ProMISe signature over all samples was used for each cancer type. All three competition models demonstrated superior performances in 9 out of the 10 cancer types while the mRNA or joint competition models achieve higher performance than the miRNA competition model (Figure 2C). Thus, ProMISe compares favorably with the existing methods, and yet has the unique advantage of constructing ProMISe signature from each individual sample.

### Cancer diagnosis

We examined whether using the ProMISe signature can discriminate tumors from normal samples in comparison with using expression profiles. We first performed hierarchical clustering as an unsupervised approach. Indeed, for each cancer type ProMISe from all three competition models cluster based on normal (blue) and tumor (red) (Supplementary Figure S5). For instance, clustering of thyroid cancer data, which consist of 58 normals and 485 tumors, is more consistent with the underlying phenotypes than clustering by expression (Figure 3B). We then performed regularized logistic regression on THCA and evaluated the testing accuracy by MCE error from LOOCV. As shown in Figure 3B, the mRNA and joint competition models achieve the lowest MCE, which is significantly better than randomized data based on permutation test (*p* < 0.01). Notably, however, the performances of each model on individual test cases tend to vary due to the heterogeneity of the tumor samples (Supplementary Figure S6). The effects of resampling on biomarker robustness and rigorous counter-strategies have been demonstrated by Li *et al.* (23). As a simple remedy, we focused our comparison on the matched tumor/normal samples to mitigate such effects in deriving tumor-specific MMIs in sections below. Together, the additional leverage provided by ProMISe over the (combined) expression data suggests that our integrative approach of modeling MMI provides useful diagnostic information complementary to the expression profile signatures. In the following analyses, we used the mRNA competition model to represent ProMISe.

### GSEA on ProMISe signature uniquely revealed abnormal activities of miR-21 and miR-145 in GBM and OV

**Figure 4.**
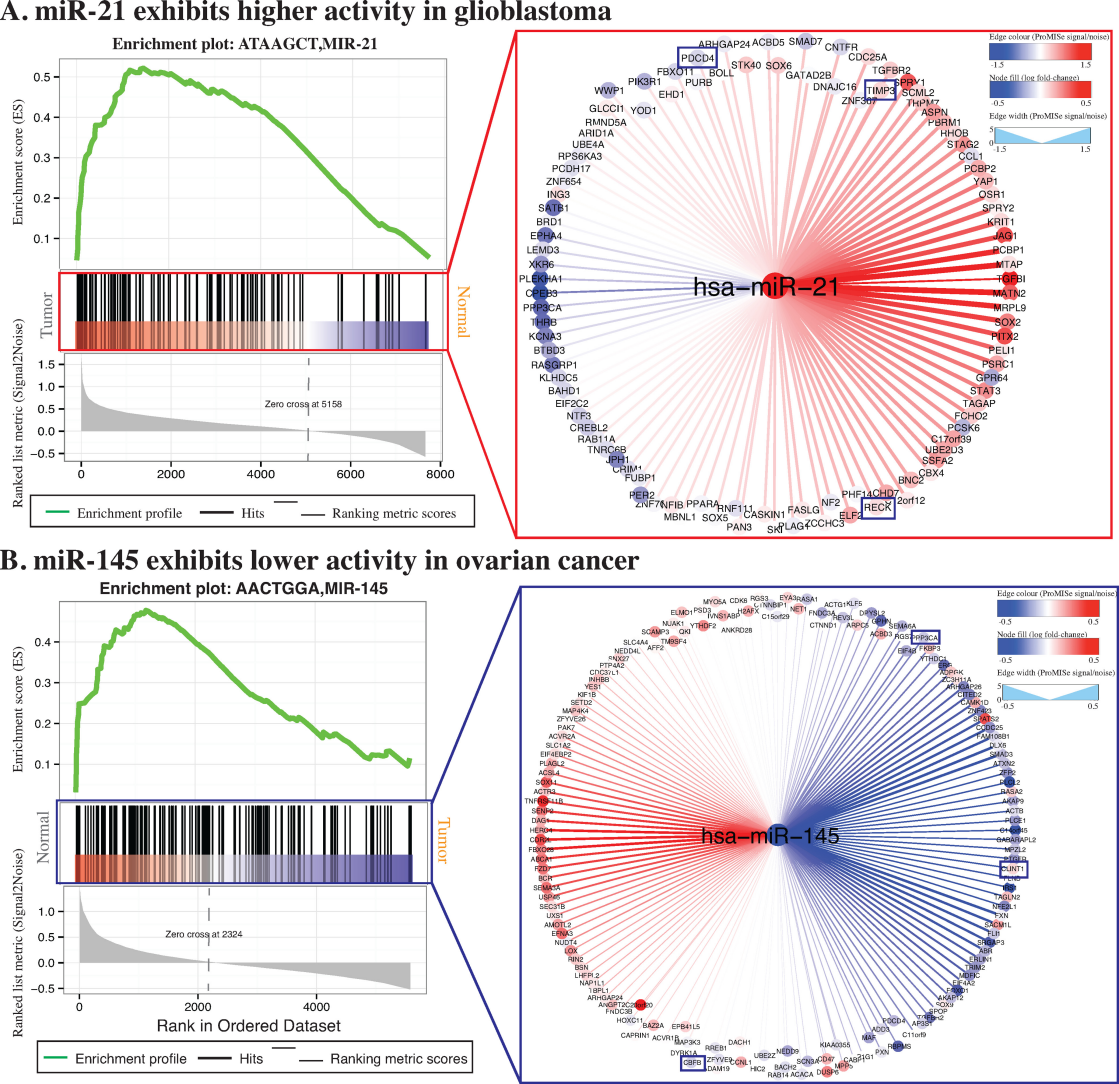
Gene set enrichment analysis. GSEA was applied to the averaged ProMISe signature of each gene over all of the miRNAs in tumor versus normal comparison (12). Enriched gene sets corresponding to miR-21 and miR-145 targets were identified from the GBM and OV data, respectively. The enriched gene sets for GBM (OV) has its gene members accumulated at the top of the list ranked by ProMISe signature changes in tumor versus normal (normal versus tumor). The right panels illustrate the network view of specific miRNA targets. The filled color in the node corresponds to expression log2 fold-change in tumor, where red (blue) indicates increased (decreased) gene/miRNA expression. The edge color and width captures the ProMISe signature changes as Signal/Noise ratio as calculated by GSEA, where red (blue) indicates increased (decreased) ProMISe signature in tumor. The network was generated using Cytoscape (26).

Aberrant miRNA and mRNA expressions have long been implicated in some specific cancer phenotypes (3). To examine whether miRNA-dysregulated genes (MDGs) are enriched for meaningful gene sets, we averaged ProMISe signature of each gene over all of the miRNAs. Surprisingly, we not only recovered cancer-specific but also oncomir-specific target gene sets with false discovery rate (FDR) <0.05 (Supplementary Table S1). In particular, synaptic transmission (MSigDB ID: M19659), psychiatric disorders (M2110), medulloblastoma (M16478), neuron cell-type specificity (M1712) and human brain aging (M9112) are among the top 10 ranked gene sets with lower ProMISe in GBM. On the other hand, gene set corresponding to the predicted miR-21 targets (M19659) ranks the second among those exhibiting higher ProMISe in GBM (Figure 4A, left). Previous studies have shown that the increased expression of miR-21 causes downregulation of tumor suppressor PDCD4, which stimulates cell growth across GBM cell lines (3,24). Indeed, we observed an increased ProMISe and a coherent decreased PDCD4 expression in tumor (Figure 4A, right). Additionally, miR-21 also regulates RECK and TIMP3 to promote anti-apoptosis and migration of GBM cells (24). Notably, both ProMISe and expression for genes RECK and TIMP3 have increased in GBM tumors, which is in fact consistent with our intuition that the higher the miRNA/mRNA abundance the more likely they interact with each other. We applied the same analysis to ovarian cancer and discovered an over-representation of miR-145 target set (M15956), ranking third among the gene sets with lower ProMISe in OV. Indeed, miR-145 is downregulated in OV and its targets confirmed by luciferase reporter assays include CBFB, PPP3CA and CLINT1 (25). Notably, CLINT1 has an increased expression and decreased ProMISe (Figure 4B)(26).

### ProMISe-predicted MDGs in tumors are enriched for canonical cancer genes and cancer genes hubs

**Figure 5.**
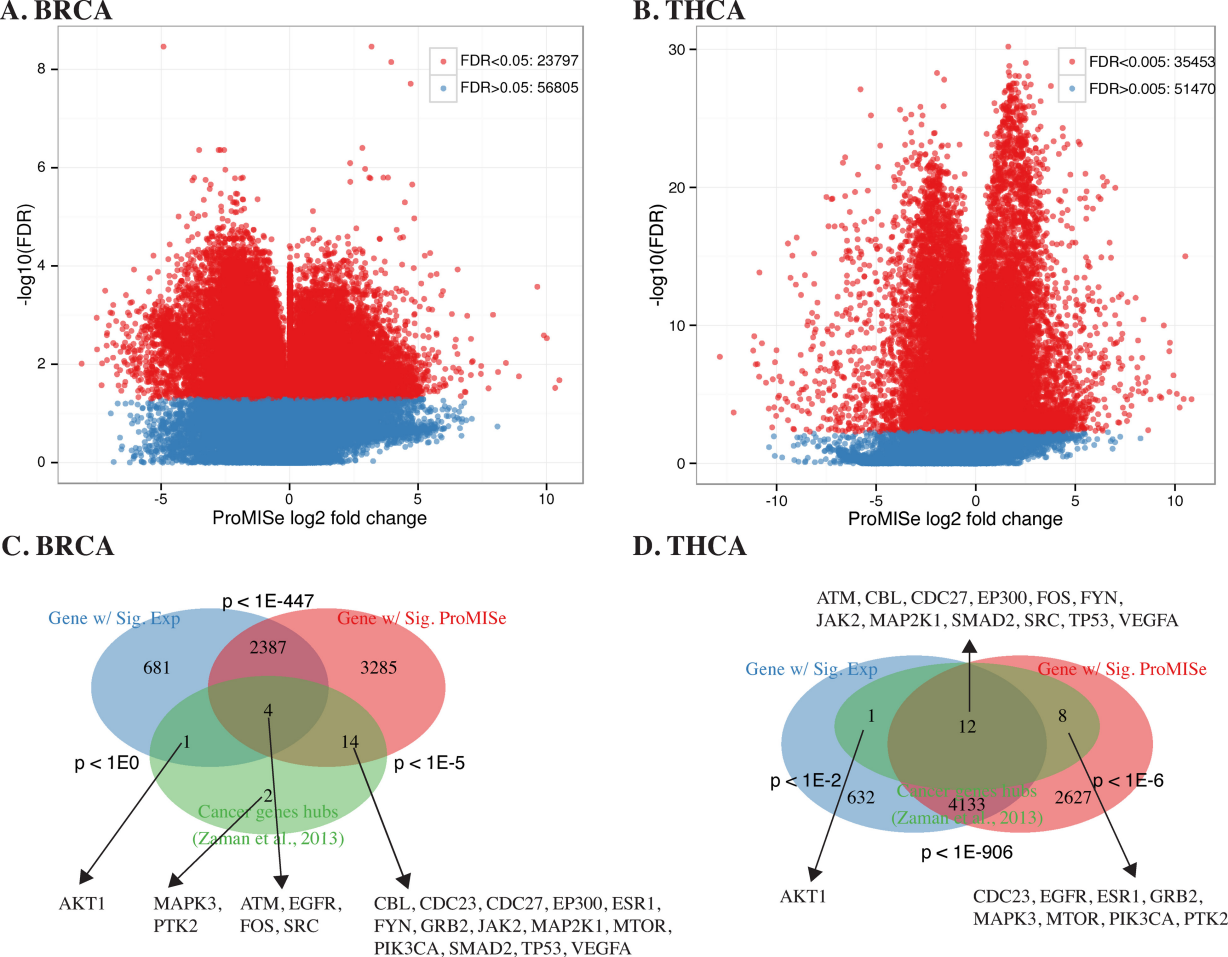
Paired sample test on matched tumor and normal in BRCA and THCA. (**A** and **B**) Volcano plots illustrate the significant interactions with −log 10(FDR) as a function of the averaged ProMISe log2 fold-change in BRCA and THCA tumors, respectively. (**C** and **D**) Three-way Venn diagram showing the overlaps among genes with differential expression (blue), genes with differential interactions (red), and putative cancer gene hubs (green) (21), respectively. Hypergeometric *p*-values are displayed near each pairwise overlap, and the common genes are displayed near the overlap.

Using the 14 matched tumor/normal samples from TCGA-BRCA data, we performed paired *t*-test to obtain differential ProMISe signature and mRNA/miRNA expression in tumor versus normal. In total, 23 797 out of 56 805 MMIs exhibit significant changes in ProMISe at FDR < 0.05 (Figure 5A), which involve 5690 out of 13035 and 103 out of 710 distinct MDGs and miRNAs, respectively. At the same cutoff, we obtained 3073 and 40 differentially expressed genes (DEGs) and miRNA. We then assessed the overlap between the DEGs and MDGs and their overlaps with the putative cancer gene hubs from (21). As illustrated in the Venn diagram (Figure 5C), the DEGs are significantly enriched for MDGs (p < 1e-447, Hypergeometric test). However, 3285 MDGs are not DEGs, and 681 DEGs are not MDGs. Moreover, MDGs are significantly enriched for cancer gene hubs: 18 out of the 21 genes have abnormal ProMISe signature in tumor (p <10^−5^, Hypergeometric test). In contrast, only 5 cancer gene hubs exhibit aberrant expression (p < 1). We repeated the same analysis on 58 matched tumor/normal samples for thyroid cancer (THCA) to obtain 35 453 out of 86 923 significant interactions at FDR < 0.005 (Figure 5B), involving 6780 (113) MDGs (miRNA) and 4778 (58) DEGs (miRNA). Twenty of the cancer gene hubs exhibit abnormal interaction with miRNAs (*p* < 10^−6^) but only 13 of them have differential expression (Figure 5D). Moreover, we compared DEGs and MDGs in both cancers with cancer-related genes from OMIM and obtained consistent results (Supplementary Figure S7). Thus, genes with differential MMIs may not have aberrant expression in tumor and vice versa. It is possible that some of the tumor-related genes are more strongly regulated at the translational levels. In that case, our approach is still able to detect at least some of these translationally regulated genes since ProMISe exploits not only the gene expression but also the miRNA expression as well as the sequence information. Moreover, the variability of gene expression profiles between individual tumors can be extremely high, which leads to low detection power of the true cancer signals using gene expression alone (23). Together, the results suggest that we have gained statistical power by identifying more cancer-related genes using ProMISe than using expression alone.

### Integrative differential analysis on breast and thyroid cancer uncovered large cancer-specific MMI network modules

**Figure 6.**
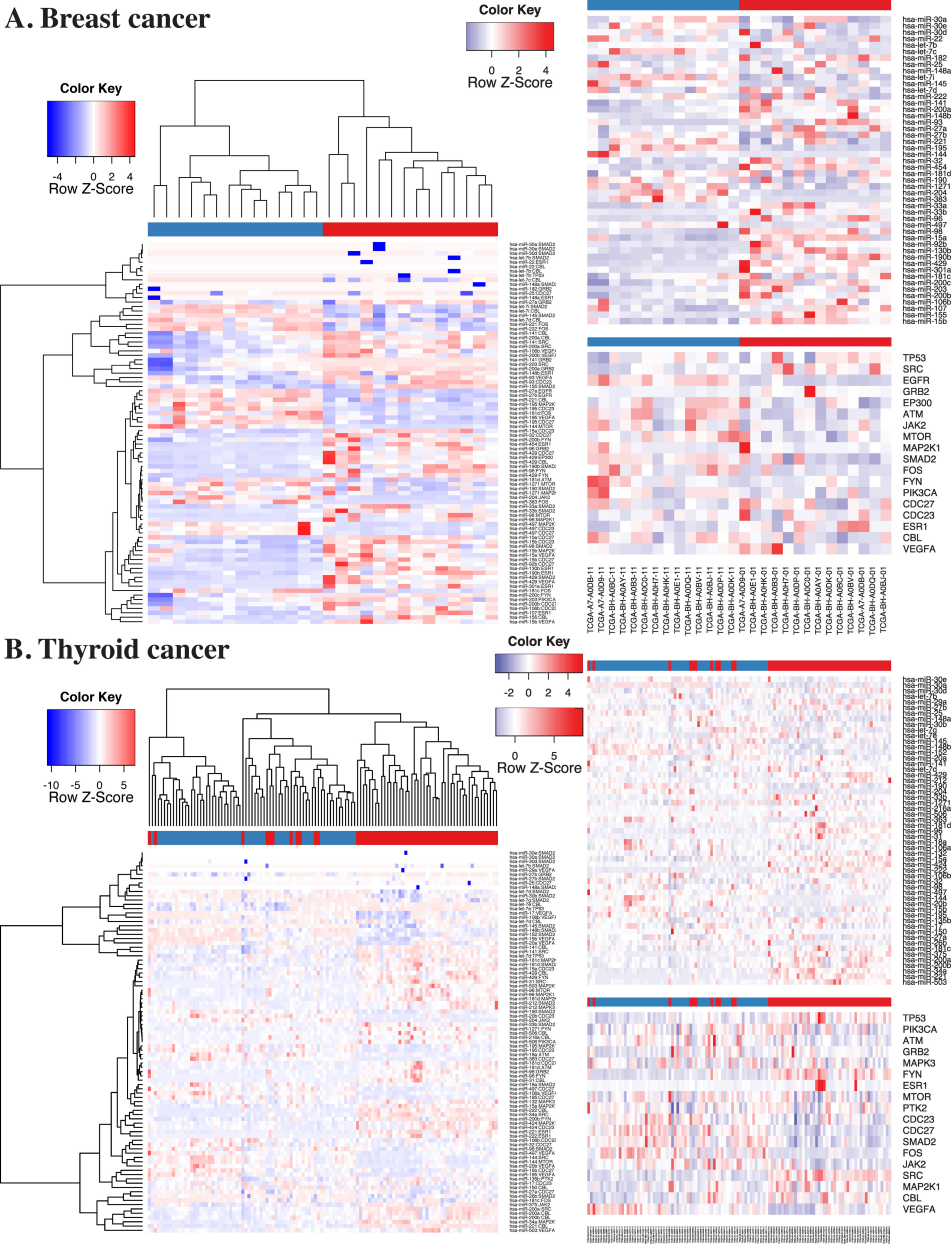
Heatmaps using differential MMIs and expression profiles for BRCA and THCA. In each panel, three sets of heatmaps corresponding to ProMISe (from the mRNA competition model), miRNA and gene expression were generated for differential MMIs involving putative cancer gene hubs from (21). Blue and red column-wise color codes for normal and tumor samples, respectively. Please refer to the main text for more details.

**Figure 7.**
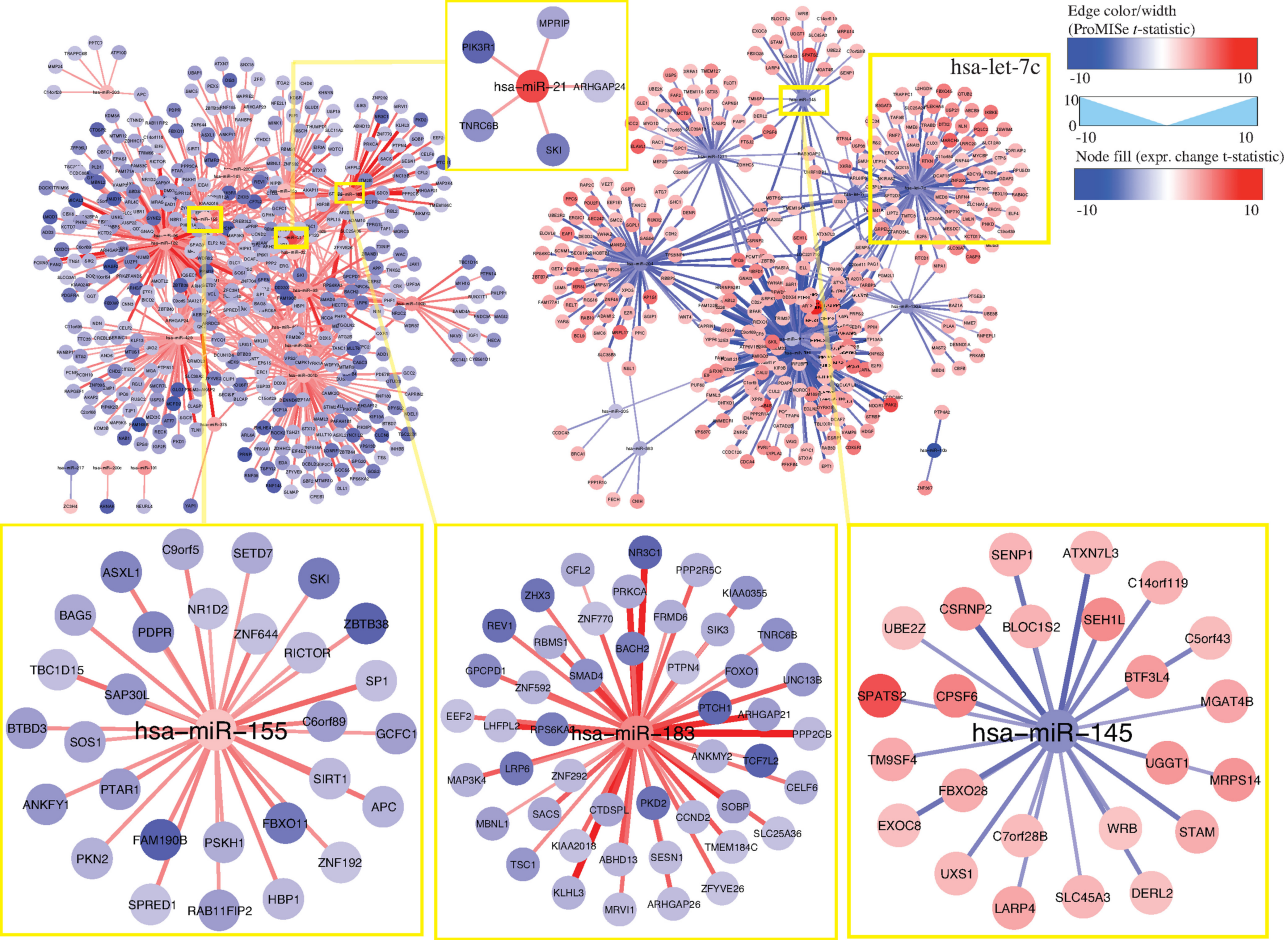
Network view of the 1257 filtered MMIs for breast cancer. The filled color in the node corresponds to *t*-statistics from paired *t*-test comparing tumor with normal, where red (blue) indicates higher (lower) gene/miRNA expression in tumor. The edge color and width captures the ProMISe signature changes in *t*-statistics, where red (blue) indicates increased (decreased) ProMISe signature in tumor. The blown-up network modules illustrate interaction changes of the BRCA-related oncomirs hsa-miR-155/21/183/145.

**Figure 8.**
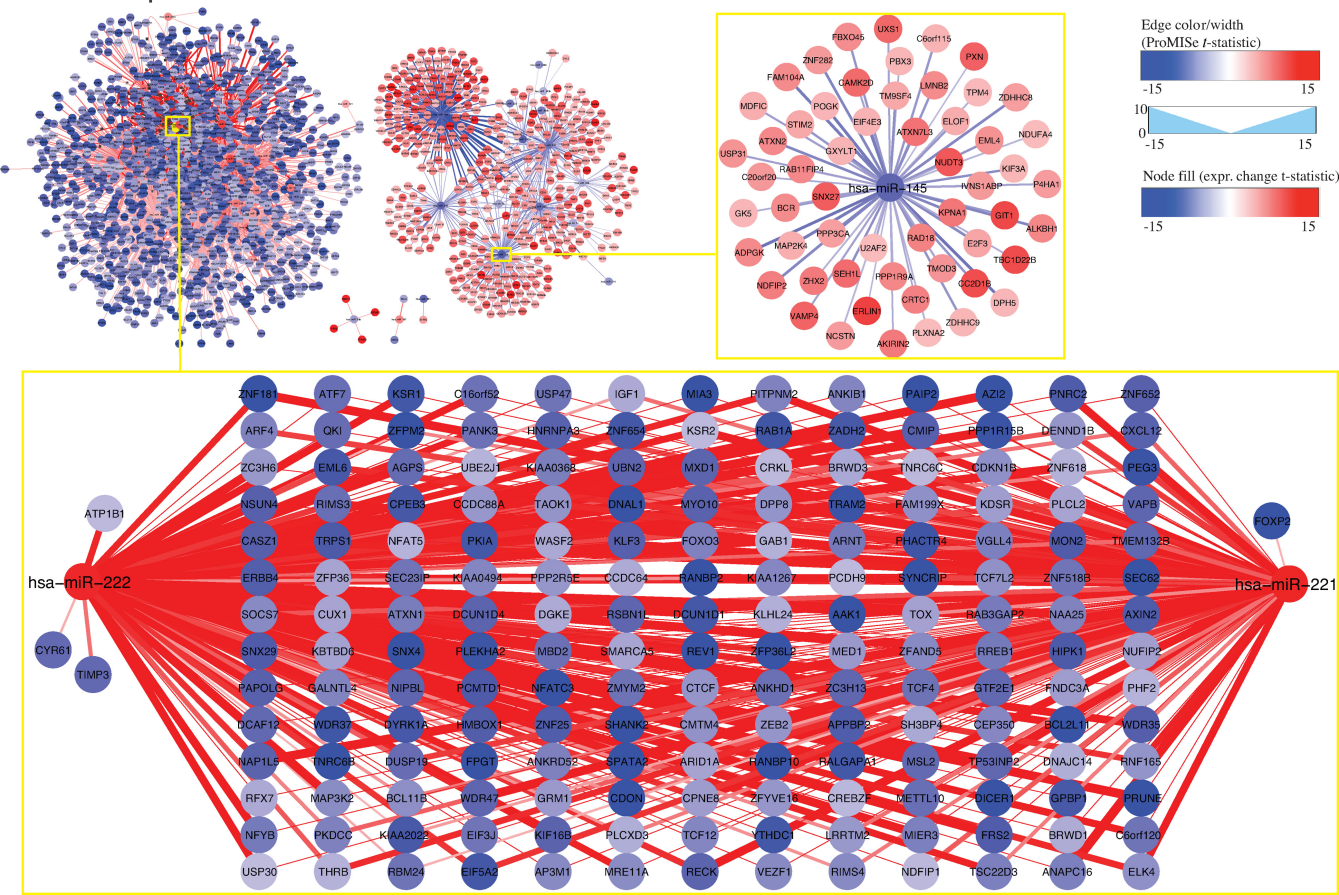
Network view of the 3255 filtered MMIs for thyroid cancer. The blown-up images display subnetworks for hsa-miR-221/222/145. Otherwise please refer to Figure 7.

To visualize tumor-related changes, we generated two sets of heatmaps for BRCA and THCA using the above differential ProMISe signature and expression profiles involving the putative cancer gene hubs (21) (Figure 6) and OMIM genes (Supplementary Figure S7). Specifically, we performed hierarchical clustering on the selected ProMISe signature profiles. As expected, normals (blue) and tumors (red) form two distinct clusters except for only a few misclassified samples in THCA. Notably, the cancer gene hubs were derived from breast cancer cell lines (21), which may explain the better clustering of BRCA samples. Interestingly, we observed a slightly more consistent clustering of THCA samples when using THCA-related genes from OMIM (Supplementary Figure S8B). The overall pattern suggests a positive correlation between the miRNA expression and ProMISe, whereas the correlation between gene expression and the ProMISe signature is less straightforward. On the one hand, genes with higher (lower) expression have higher (lower) likelihood of being targeted. On the other hand, decreased (increased) MMIs may ultimately imply an increased (decreased) gene expression. Accordingly, we devised the following rule to retain only a subset of the tumor-specific interaction changes coherent with expression changes: (i) the tumor-specific differences in terms of ProMISe signature, gene and miRNA expression must be significant; (ii) the change sign between ProMISe signature and miRNA must be the same; (iii) the change sign between ProMISe signature/miRNA and gene expression must be the opposite. Some of the qualified pairs are highlighted in purple boxes in Supplementary Figure S8. In total, we obtained 1257 and 3255 qualified interactions for BRCA and THCA, corresponding to 748 (32) and 1679 (44) distinct genes (miRNAs), among which 42 (9) and 77 (15) are oncogenes (oncomirs), respectively (Supplementary Table S2). The restricted MMI forms two large network modules, harboring genes with significant up- (red) and downregulation (blue) status associated with decreased (blue edge) and increased (red edge) ProMISe signature, respectively (Figures 7 and 8). For instance, the increased expression of hsa-miR-155/21/183 is coupled with increased interactions with its target genes, which exhibit decreased expression in breast tumor samples (Figure 7). Previous studies have shown the expression increase of the three miRNAs in breast cancer tissues or cell lines; however, their targetomes specific to breast cancer are not well characterized (3). On the other hand, BRCA-related oncomirs including let-7c and miR-145 are underexpressed in BRCA (Figure 7). Both let-7c and hsa-miR-145 are tumor suppressor genes and modulate motility, inhibit cell growth, and induce apoptosis of breast cancer cell lines (3,27). For thyroid cancer, our results remarkably show that hsa-miR-221/222 are overexpressed, consistent with the previous finding (3), and significantly downregulate a large cohort of genes including oncogenes ARID1A, ARNT, BCL11B, DICER1, ELK4, TCF12, TCF7L2, ARID1A, ARNT, BCL11B, DICER1, ELK4, TCF12 and TCF7L2 (Figure 8). Notably, miR-145 is also downregulated in THCA coupled with upregulation of oncogenes BCR, EML4, MAP2K4 and TPM4. Thus, the reduced activities of miR-145 are prevalent in breast, ovarian and thyroid cancers based on our results, perhaps underlining its important role in maintaining normal cell state. Together, our novel integrative approach enables discovering cancer-specific miRNAs and associating them with oncogenes at system level.

## DISCUSSION

The existence of cell-type-specific expression ‘signatures’ of miRNA and gene has been previously appreciated (28). However, to our knowledge, there is no systematic method that identifies the MMI signature from each individual sample. In particular, most existing methods are based on negative correlation (5,29,30,31,32). These methods can only identify MMIs by aggregating multiple samples. In a real-world application such as cancers, however, the correlation-based methods are limited to identifying only the robust interactions due to the heterogeneity of individual expression profiles. Additionally, most existing methods are in a regression framework, by treating mRNAs as response and miRNAs as input variables, which assumes that mRNAs are independent of one another. However, mRNAs targeted by a common miRNA ‘do’ interact by competing for that miRNA (9). In this study, we describe a novel approach and a new class of molecular signature collectively termed as ProMISe. In essence, ProMISe signature is an *N* × *M* probability matrix for interactions between mRNA *i* and miRNA *k*. Notably, we do not normalize the probabilities so that each row or column sums to 1 because a miRNA can target multiple mRNAs with high probabilities and vice versa. Also, miRNA members in the same family (i.e. miRNAs with the same seed region) may be expressed differently. Thus, we keep them separate rather than combining them to reflect the competition within as well as between miRNA families. We explored three model formalisms, namely ‘mRNA competition’, ‘miRNA competition’ and ‘joint competition’. Although all three models compare favorably with the existing methods, our results more strongly support the mRNA competition model. Presumably, the mRNA competition has more prevalent effects on MMI because there are more potential mRNA targets per miRNA than there are miRNA regulators per mRNA.

The success of our approach is based on the very fact of the miRNA targeting mechanism: each miRNA molecule can only bind to and induces degradation of one mRNA molecule at a time. This concept fits nicely with the digital read count offered by the next-generation sequencing technologies. In contrast, the same technique is not readily applicable to model transcript factor (TF)–gene interactions since the enzymatic activities of each TF can differ widely regardless of its physical abundance at mRNA or protein level. On the other hand, we also made several simplifying assumptions. First, we assume that mRNA without any seed match to a particular miRNA will have zero probabilities of being targeted by that miRNA. Recent literatures suggest a ‘seedless’ model, where miRNA can bind to regions other than 3′UTR with imperfect matches (33). Inclusion of seedless matches, however, will introduce a large number of false positives and thus requires a more specific model. Second, we assume the binding of miRNA will always induce the mRNA degradation, which is supported by a recent study via ribosome profiling after miRNA perturbations (34). However, some miRNAs may modulate gene expression primarily at the translational level (35,36). In that case, only the seed-match matrix and miRNA expression are informative to inferring MMIs. By comparing observed mRNA levels with the proteomic data (if available), we should be able to more realistically capture the mode of translational repression. Third, we assume the binding efficacy is the same between any miRNA and any mRNA. It is more realistic to use different mRNA–miRNA binding efficacy, which can be estimated from miRNA over- or underexpression data (4). Fourth, we do not know the grand total amount of transcripts within the cellular capacity under a specific condition. In our model, we arbitrarily assume that the total *T* is 30% (by default) more than the observed total. Fixing the total amount prevents ever-increasing individual total RNA levels estimated in each iteration but introduces a free parameter into the model. Since the miRNA targeting primarily occurs in the cytoplasm, it may be possible to estimate the total amount *T* by simultaneously measuring the RNA in both nucleus and cytoplasm using recently developed RiboMinus RNA-seq technology (37). Fifth, we only consider mRNAs as competitors for miRNAs in our model. Several recent studies have focused on the interplays between miRNA and long noncoding RNA (lncRNA) including pseudogene (38) as well as circular RNA (circRNA) (39), which are collectively termed as the competing endogenous RNA (ceRNA). Thus, it would be more realistic to consider the seed match and abundance of ceRNA when inferring MMI. Finally, there are many other factors that we have not considered, which nonetheless influence the outcome and our interpretation of MMI. For instance, our model only considers the total expressed seed matches at 3′UTR of mRNA for the same miRNA in the mRNA competition model and total expressed seeds of miRNA for the same mRNA in the miRNA competition model. It would be more informative to examine single nucleotide polymorphism in tumor samples, which may disrupt (introduce) existing (new) seed regions on miRNAs and/or seed matches on mRNAs. Moreover, aberrant expressions may merely be the consequence of copy number variation (CNV) at the genomic DNA level (21), which are not directly related to miRNA dysregulation. In tumors, some mRNAs may be regulated transiently by TF and/or miRNA, which may only be captured by single-cell RNA-seq (40). With more data becoming available from TCGA and elsewhere, we will examine these possibilities in future work.

Comparing with other methods, the most distinct feature of ProMISe is its ability to operate on a single pair of miRNA and mRNA expression profiles measured from the same individual. This unique ability allows us to construct ProMISe signature from each individual tumor or normal sample, perform cancer diagnosis using this novel molecular signature (Figure 3), and ultimately predict tumor-specific MMIs (Figures 7 and 8). For the latter, we focused our analysis on only the respective 14 and 58 matched tumor and normal samples in BRCA and THCA to mitigate heterogeneity among cancer samples. However, a significantly higher (lower) likelihood of MMI in tumor (w.r.t. normal) may merely reflect the significantly higher (lower) expression of miRNA and/or gene, which may in turn be the consequence of CNV (21). Although we show that genes involved in aberrant MMIs are enriched for meaningful oncogenic signature genes (Figure 5), it is important to distinguish two scenarios: (i) aberrant gene expression is caused by miRNA dysregulation; (ii) abnormal MMI reflects aberrant miRNA/gene expression. We designed a simple way to minimize the confounding effects caused by the second scenario. In particular, we focused only on the significant interactions with the same change sign as miRNA expression change but opposite change sign to the gene expression changes. Surprisingly, we were still able to obtain as many as 1257 and 3255 qualified interactions for BRCA and THCA, respectively (Supplementary Table S2). Visualizing these interactions in the network context revealed several meaningful network modules involving previously discovered and potential oncomirs and oncogenes (Figures 7 and8). From clinical perspective, the decreasing cost of genome-wide large and small RNA profiling will facilitate designing personalized medicine in small RNA therapy. Ideally, the unique ProMISe signature inferred from the paired expression profiles from an unknown sample will be compared with the categorized ProMISe signature established from existing data to identify disease-specific miRNA regulatory network modules. The knowledge link between miRNA and mRNA will enable designing drugs that control both the aberrant miRNA and gene expression synergistically.

## SUPPLEMENTARY DATA

Supplementary Data are available at NAR Online, including [1–6].

SUPPLEMENTARY DATA
