# Supplementary material for: Inferring probabilistic miRNA–mRNA interaction signatures in cancers: a role-switch approach
Source: Nucleic Acids Res. 2014 Mar 7;42(9):e76. doi: 10.1093/nar/gku182 (PMC4027195; doi:10.1093/nar/gku182)
Supplement: SUPPLEMENTARY DATA [file supp_42_9_e76__index.html]

Inferring probabilistic miRNA–mRNA interaction signatures in cancers: a role-switch approach — SUPPLEMENTARY DATA 

# Inferring probabilistic miRNA–mRNA interaction signatures in cancers: a role-switch approach

## SUPPLEMENTARY DATA

**Files in this Data Supplement:**

- Supplemental Figures
- Supplemental Table
- Supplemental Tables
