# Supplementary material for: Inferring probabilistic miRNA–mRNA interaction signatures in cancers: a role-switch approach
Source: Nucleic Acids Res. 2014 Mar 7;42(9):e76. doi: 10.1093/nar/gku182 (PMC4027195; doi:10.1093/nar/gku182)
Supplement: SUPPLEMENTARY DATA [file supp_gku182_nar-03694-met-n-2013-File018.pdf]

Published online XX XXX XXXX *Nucleic Acids Research, XXXX, Vol. XX, No. XX* ***1–9***
doi:10.1093/nar/gkn000

Inferring probabilistic miRNA-mRNA interaction signatures in cancers: a role-switch approach

Yue Li [1,2], Cheng Liang [2] Ka-Chun Wong [2] Ke Jin [2] Zhaolei Zhang [1,2,3,4,*]

[1]Department of Computer Science, [2]The Donnelly Centre, University of Toronto, Toronto, ON, M5S 3E1, Canada, [3]Department of Molecular Genetics, University of Toronto, Toronto, ON, M5S 1A4, Canada and [4]Banting and Best Department of Medical Research, University of Toronto, Toronto, ON, M5S 3E1, Canada

Received X X, XXXX; Revised X X, XXXX; Accepted X X, XXXX

SIMULATION

We simulated for 1000 mRNAs and 20 miRNAs their true expression from Gaussian distribution using `rnorm` with mean and standard deviation set to 3 and 1, respectively. To gain a robust estimate, the simulations were repeated 10 times and the averaged values were taken. The 1000×20 seed matrix were generated (using `rpois`) from a Poisson distribution with $\lambda=0.2$. The true miRNA-mRNA interactions were simulated by requiring the probabilities computed by Eq **1**, **2**, and **3** to be all greater than the respective 90% quantiles. Unless mentioned otherwise in the following tests, we added standard normal noise to the true miRNA and mRNA expression to represent the observed miRNA and mRNA expression and one false seed match to 60% of the seed matches. The added noise and false positive seed matches were intended to reflect the real situation, where the measurements (RNA-seq or microarrays) are noisy and the information for the miRNA recognition sites are not perfectly accurate. To examine the model behaviours and compare the three competition models, we designed four scenarios outlined below:

1. We tested the robustness of each above methods by increasing noise levels as the Gaussian means from 1 to 3 with 0.5 increments to the observed miRNA and mRNA expression values. At each noise level, we repeated the tests 100 times (Fig. S2A).

2. Similar to test 2, we increased the percent of false positive seed matches by adding one false positive to 0%-80% of the seed matches with increment of 20%. At each false positive rate, we repeated the tests 100 times (Fig. S2B).

3. We examined whether each model performs better in fully observed data comparing with partially observed data by randomly setting zero to $x\%\in\{0\%,\ldots,80\%\}$ (with increment of 20%) of the observed data, where $x\%=0\%$ (80%) means 100% (only 20%) observed. At each partially observed level, we repeated the tests 100 times (Fig. S2C).

4. We examined whether averaging the model predictions (Eq **3**) over replicates improves the model performances. We created $R\in\{40,20,10,5,2,1\}$ replicates from the simulated data by adding default random noise to each replicate. We then applied each model to the R replicates separately. At each replicate number, the tests were repeated 100 times (Fig. S2D).

For each test in each scenario above, we assessed the sensitivity and specificity of the methods using ROC curve and summarized the performances by the area under the curve (AUC). For a given score cutoff, the true and false positive rates (TPR and FPR) are estimated as the respective ratios of TPR=TP/P and FPR=FP/N, where TP and FP are the numbers of true and false positives, and P and N are the total numbers of positive and negative miRNA targets in the test data. ROC is plotted by iteratively evaluating TPR (y-axis) and FPR (x-axis) while relaxing the scoring cutoff. In addition, we constructed precision-recall (PR) curve (PRC) and assessed the precision TP/(TP+FP) of each model at the same recall (or TPR). Both ROC and PR statistics were obtained using *ROCR* package (1).

*To whom correspondence should be addressed. Tel: +1 (416) 946-0924; Fax: +1 (416) 978-8287; Email: zhaolei.zhang@utoronto.ca

© XXXX The Author(s)
This is an Open Access article distributed under the terms of the Creative Commons Attribution Non-Commercial License (http://creativecommons.org/licenses/by-nc/2.0/uk/) which permits unrestricted non-commercial use, distribution, and reproduction in any medium, provided the original work is properly cited.

SIMULATION RESULTS

To rigorously test the models, we evaluated each model based on the four scenarios described above each testing an important property of the models. Fig. S2 summarizes the simulation results. Overall, the mRNA competition and joint competition models achieve comparable performances that compare favourably with the miRNA competition model and the seed matrix. First, all three competition models are robust to increasing levels of noise on the observed mRNA and miRNA expression (Fig. S2A). Thus, the proposed model is suited for high-throughput platforms such as microarrays and RNA-seq, which usually produce noisy expression measurements. Second, on the other hand, all of the tested models are sensitive to increasing percentages of false seed-matches (Fig. S2B). The result is expected since all of the target predictions are essentially derived from the seed-match. Third, the three models perform better on fully observed data than partially observed miRNA/mRNA expression (Fig. S2C). The result confirms the expected model behaviour, which is to exploit all of the mRNA/miRNA expression (as provided by RNA-seq or microarray technologies) to infer each individual targeting relationship (Eq **1**,**2**). Consequently, when the expression of the competitive mRNAs j and/or miRNA l are not measured, the model will not be able to predict accurately on the interaction between mRNA i and miRNA k ($i \neq j$ or $k \neq l$). We emphasize that this is not a limitation of the model since high-throughput platforms such as microarrays and RNA-seq provide genome-wide expression profiles of miRNA and mRNA. Finally, the models perform better with increasing number of replicates by averaging the prediction scores (Fig. S2D). Thus, the model is able to take advantage of the fact that most expression profiling experiments are performed in replicates to deliver more reliable prediction. Below we compared ProMISe with other methods using real expression data.

SUPPLEMENTARY FIGURES

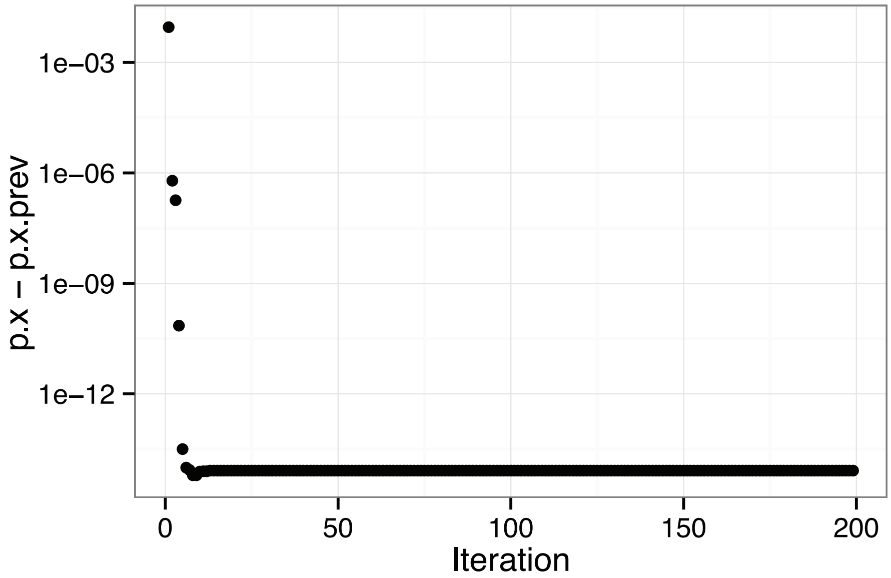

Figure S1. Model convergence. Expression of 20000 genes and 400 miRNA were simulated from Gaussian distribution with mean equal to 3 and standard deviation equal to 1. ProMISe was applied to the expression profiles with convergence threshold tol set to 1e-20. The progress in terms of the maximum absolute difference between the probabilities of miRNA-mRNA at the i and $i-1$ iteration (Eq **1** in *Materials and methods*) as a function of iterations was recorded. The above plot was generated by averaging the results from 100 repeated runs.

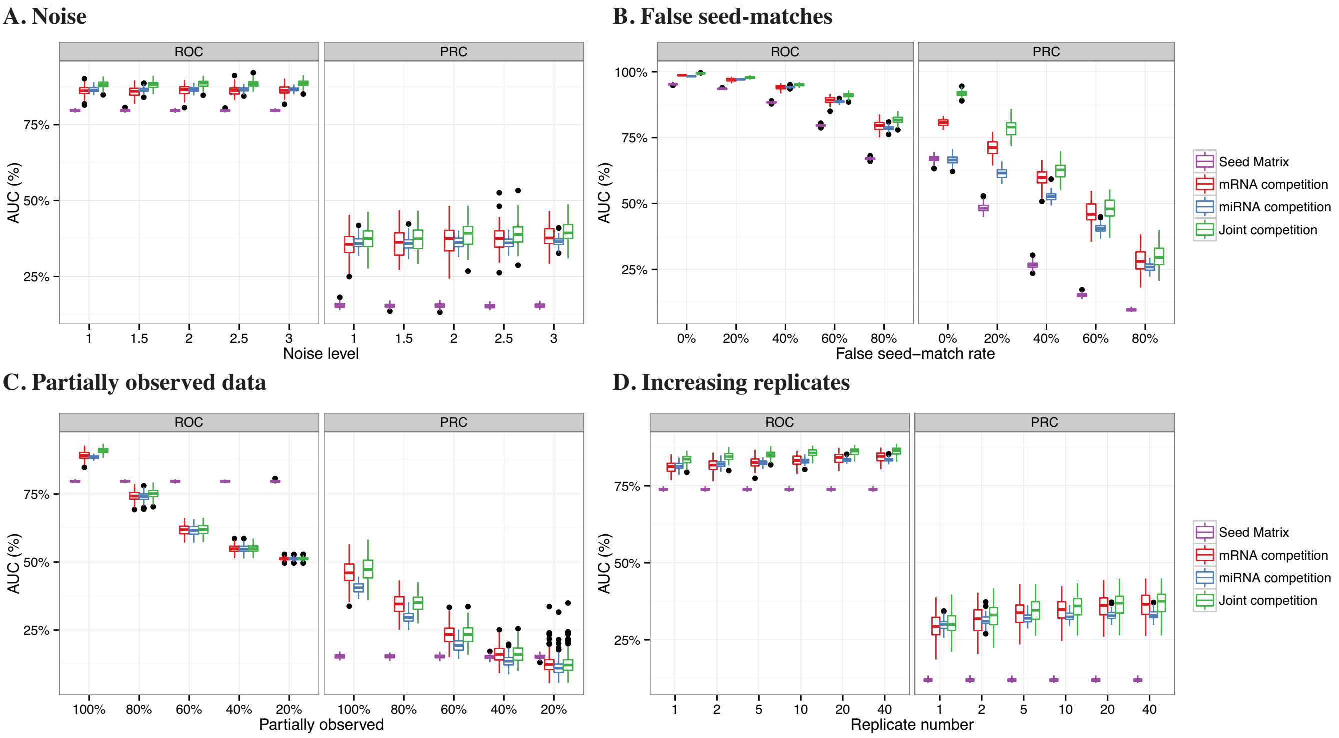

Figure S2. Simulation. The true expression of 1000 mRNAs and 20 miRNAs were simulated from Gaussian distribution with mean and standard deviation equal to 3 and 1, respectively. The 1000×20 seed-match matrix was simulated from Poisson with 0.2 rate. Unless mentioned otherwise, standard normal noise was added to the expression to mimic the noisy measured expression, and one false seed match was added to 60% of the seed-match matrix entries to mimic the imperfect target site information. Seed-match matrix was used as the baseline model, which considers the higher the number of target sites c_{ik} the more likely mRNA i to be the target of miRNA k, regardless of their expression levels. mRNA competition, miRNA competition, joint competition correspond to three proposed model formulations (*Materials and methods*). **A** & **B**. evaluation of robustness in terms of AUC with increasing noise levels (i.e. the means of Gaussian noise) on both miRNA and mRNA expression, and increasing percentages of false seed-matches; both tests were repeated 100 times and boxplots of AUC were drawn; **C** & **D**. influence of degrees of partially observed data and increasing number of replicates on prediction accuracy of the proposed models.

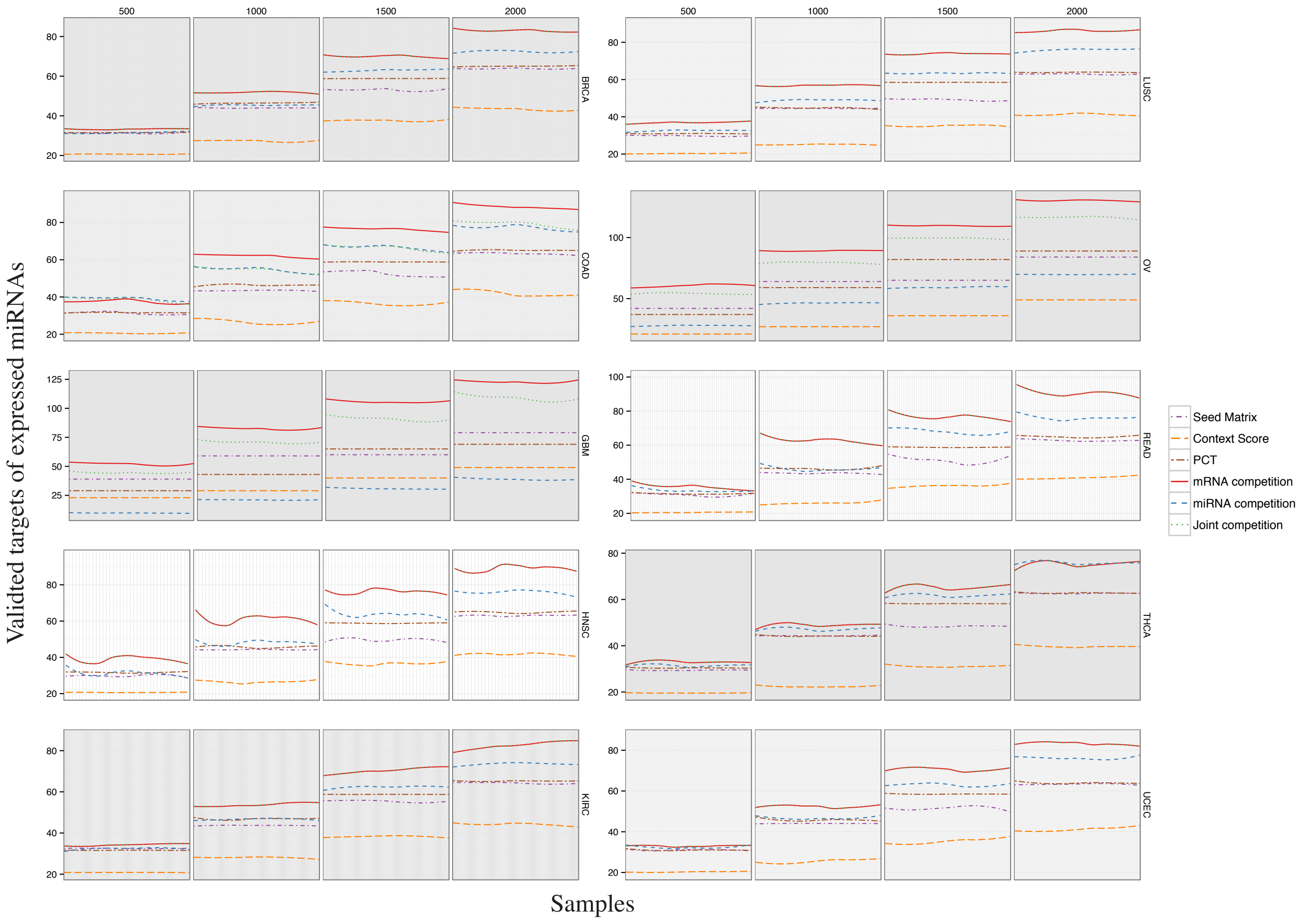

Figure S3. ProMISe vs sequence-based methods. For each cancer type, we counted the number of validate targets among the top 500-2000 ranked targets by Seed Matrix (i.e., number of conserved sites), Context Score and PCT (probability of conserved targeting) and the three proposed competition models. All three sequence-based matrices are predicted by TargetScan (2, 3). Samples are sorted by their ID (x-axis omitted). The y-axis displays the number of validated targets based on MirTarBase (4) among the top N rank from each method. The 10 panels represent the cancer types from TCGA data. For clarity, the curves are smoothed using `loess` method from `geom_smooth`.

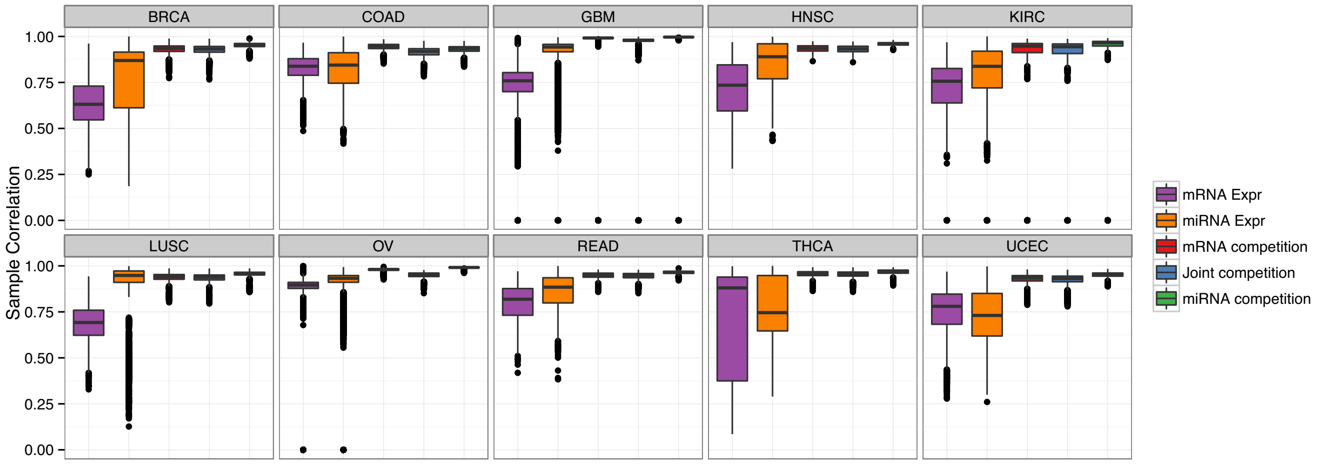

Figure S4. Sample correlation in terms expression profiles or ProMISe signature. Pairwise correlation between distinct pairs of samples (i.e., the upper/lower triangle of the symmetrical correlation matrix) were displayed as boxplots for gene/miRNA expression profiles and ProMISe calculated by mRNA competition, miRNA competition, or joint competition models.

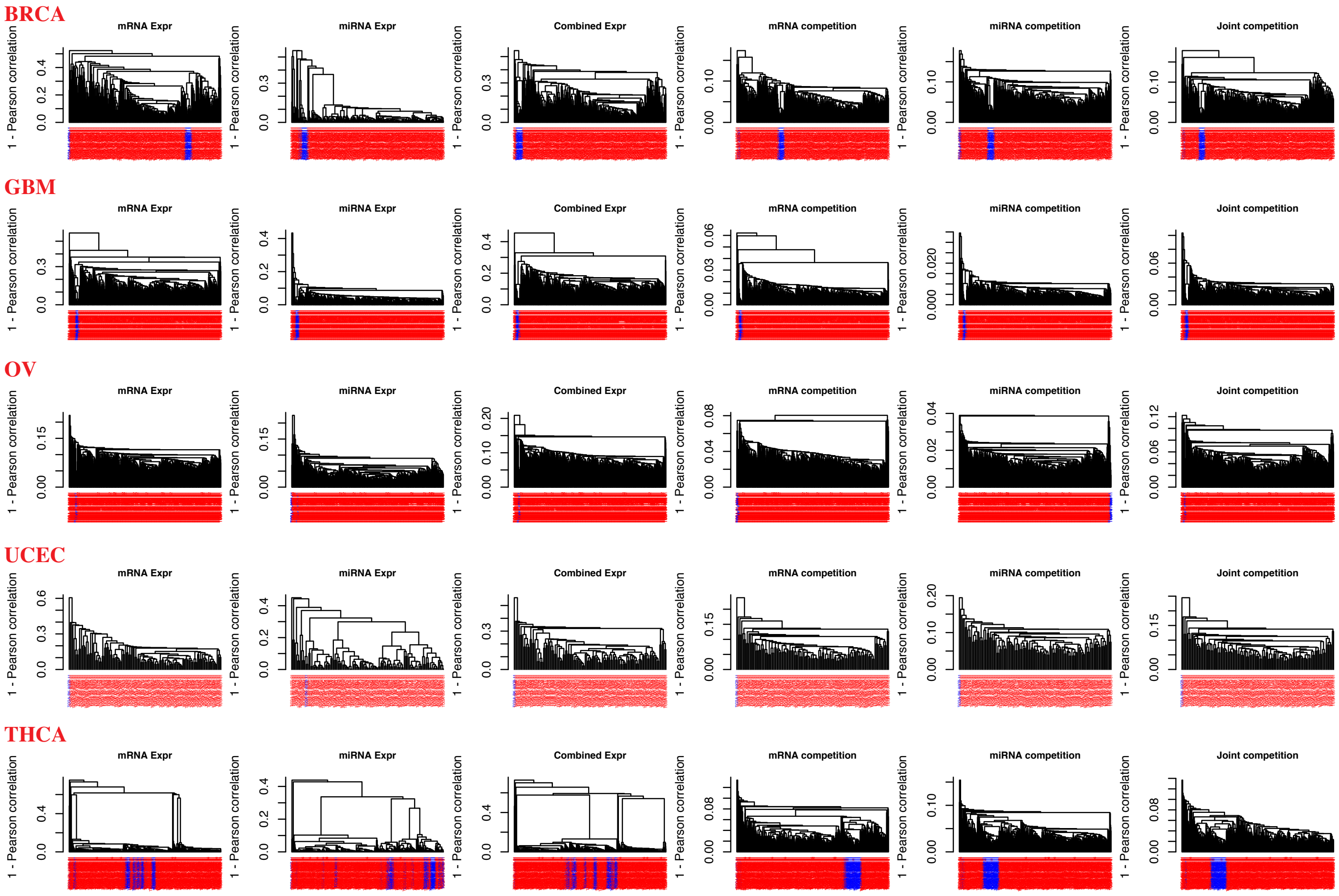

Figure S5. Hierarchical clustering Hierarchical clustering of gene expression, miRNA expression profiles, combined expression profiles, and ProMISe from the three competition models. Red and blue-coded colour indicates the tumor and normal samples from selected cancers with at least one normal from the TCGA data.

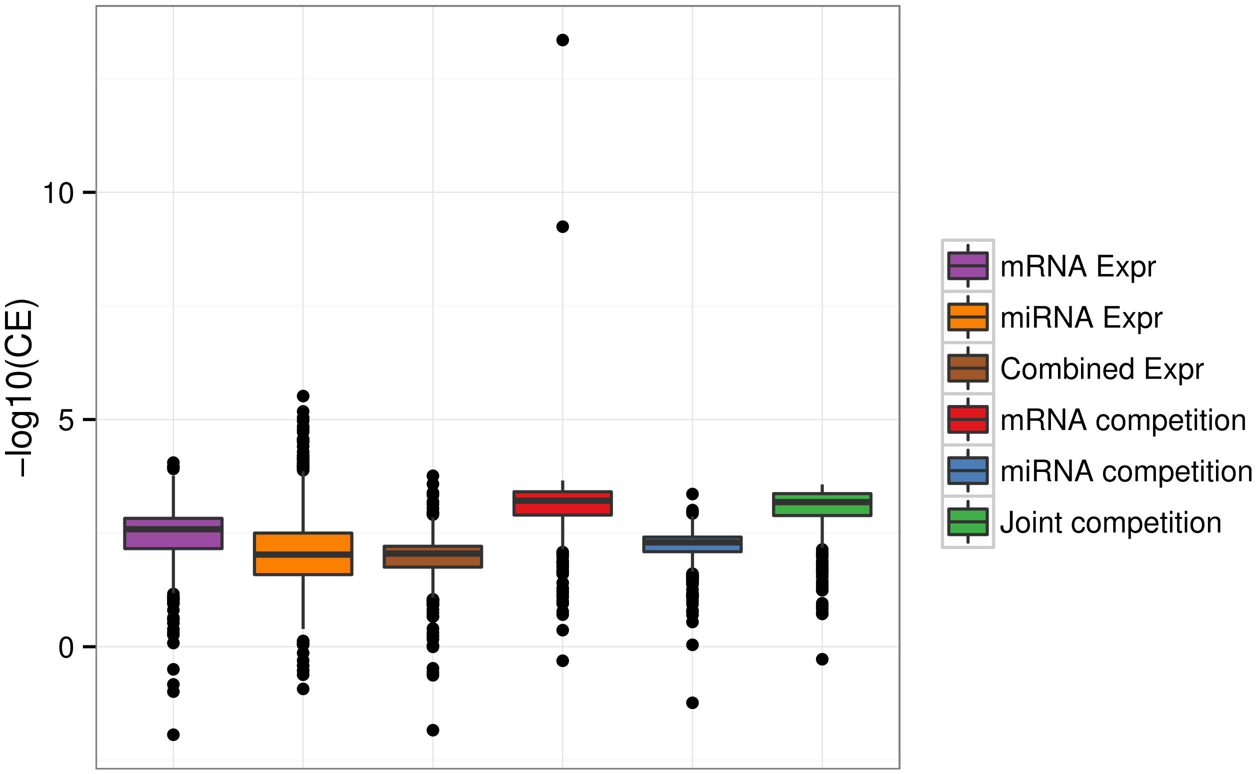

Figure S6. Cancer diagnosis. Regularized logistic regression was applied to classify normal and thyroid cancer tumor profiles using expression or ProMISe signature. Cross entropy (CE) from LOOCV was used to assess the performance of each method. Superior method confers lower CE and thus higher -log10(CE).

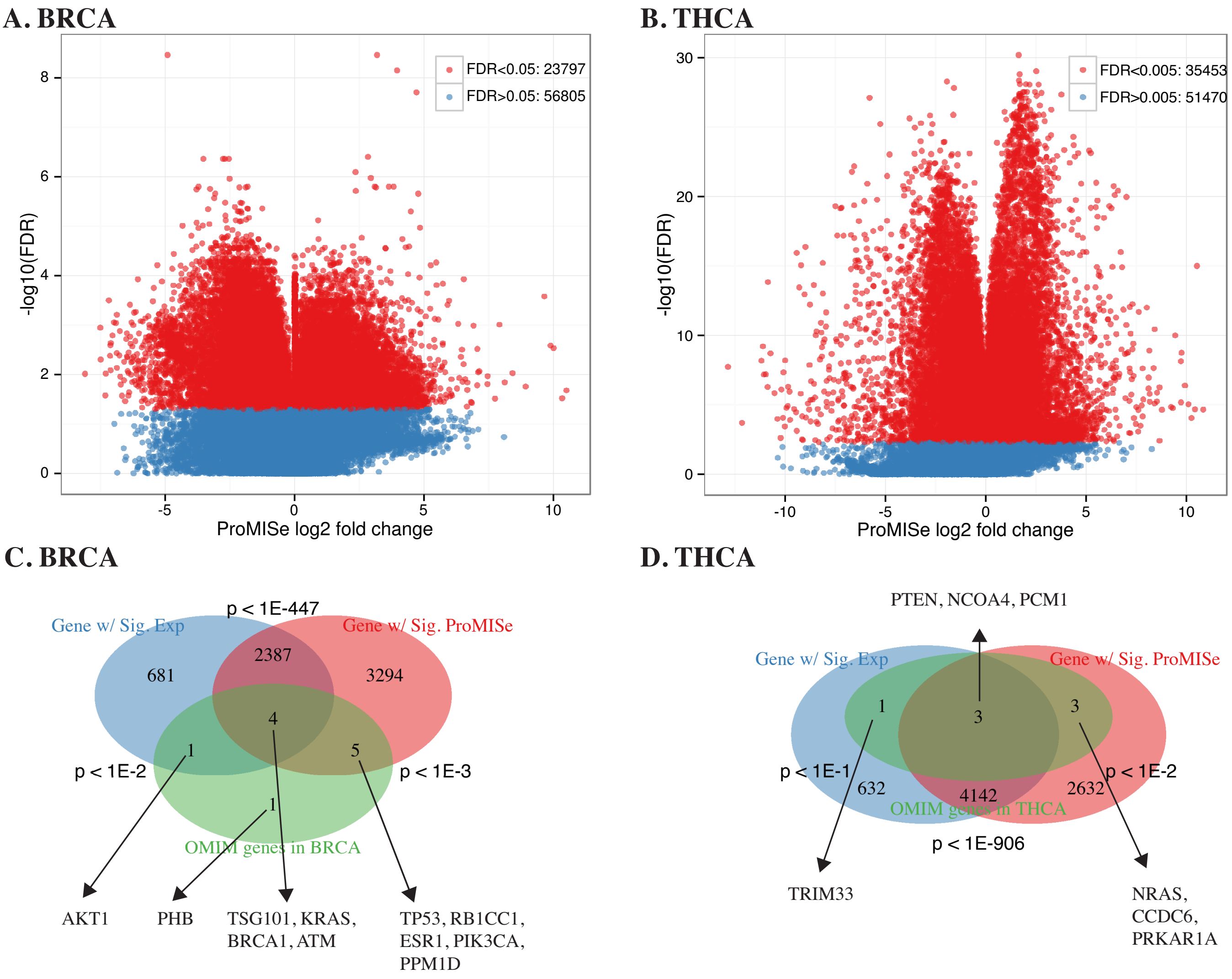

Figure S7. Paired sample test on matched tumor and normal in BRCA and THCA. **A & B**. Volcano plots illustrate the significant interactions with $-\log 10(\text{FDR})$ as a function of the averaged ProMISe log2 fold-change in BRCA and THCA tumors, respectively. **C & D**. Three-way Venn diagram showing the overlaps among genes with differential expression (blue), genes with differential interactions (red), and BRCA and THCA-related genes from OMIM, respectively. Hypergeometric p-values are displayed nearby each pairwise overlap, and the common genes are displayed nearby the overlap.

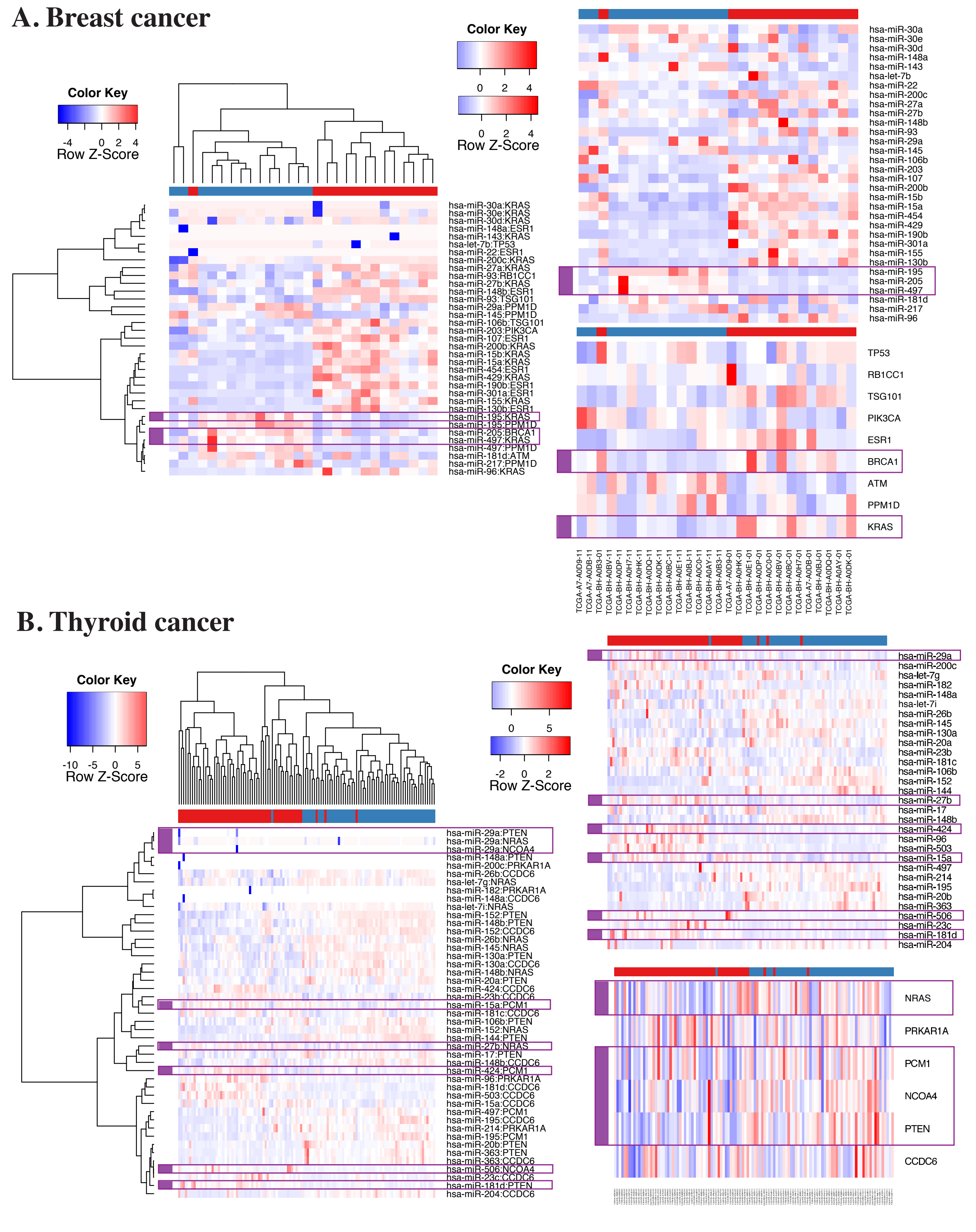

Figure S8. Heatmaps using differential MMI and expression profiles for BRCA and THCA In each panel, three sets of heatmaps corresponding to ProMISe (from the mRNA competition model), miRNA and gene expression were generated for differential MMI involving cancer-specific genes from OMIM. Blue and red column-wise colour codes for normal and tumor sample, respectively. Rows highlighted by purple boxes are the MMI involving coherent expression changes of miRNA and the corresponding targets. Please refer to the main text for more details.

SUPPLEMENTARY TABLES

Table S1.

Top 100 enriched gene sets from GSEA on ProMISe and gene expression on the BRCA, GBM, OV, THCA, and BRCA. ProMISe were averaged over all miRNA to represent the overall miRNA regulation per gene. Column 1-5: status that indicates whether the ProMISe or expression is higher or lower in tumor, gene set names, normalized enrichment score, nominal p-value, and false discovery rate (FDR) after multiple testing correction.

Table S2.

Paired test on matched tumor and normal samples from BRCA/THCA data. miRNAs and genes in each miRNA-mRNA interaction pair are listed in separate columns. Columns 1 and 2 list the miRNA and gene names. Columns 3-8 are averaged ProMISe and the differences between normal and tumor, t-statistics, and p-value from the paired t-test on quantile normalized ProMISe, and adjusted p-value (qval) by BH method. Columns 9-12 indicates whether the interaction has been validated based on MirTarBase, corresponding gene is a oncogene based on Cosmic, corresponding miRNA is a oncomir based on (5), and whether both the pair involves both oncogene and oncomir. Columns 13-22 display the gene and miRNA averaged expression and the t-statistics form paired t-test. Column 23 indicates whether the interaction qualifies the criteria discussed in the main text. Column 24 indicates whether the gene is a BRCA/THCA-related genes from OMIM. Column 25 indicates the putative cancer hub genes from (6).

REFERENCES

1. Sing, T., Sander, O., Beerenwinkel, N., and Lengauer, T. (2005) ROCR: visualizing classifier performance in R. *Bioinformatics,* **21**(20), 7881.
2. Friedman, R. C., Farh, K. K.-H., Burge, C. B., and Bartel, D. P. (January, 2009) Most mammalian mRNAs are conserved targets of microRNAs.. *Genome Research,* **19**(1), 92–105.
3. Garcia, D. M., Baek, D., Shin, C., Bell, G. W., Grimson, A., and Bartel, D. P. (October, 2011) Weak seed-pairing stability and high target-site abundance decrease the proficiency of lsy-6 and other microRNAs.. *Nature structural & molecular biology,* **18**(10), 1139–1146.
4. Hsu, S.-D., Lin, F.-M., Wu, W.-Y., Liang, C., Huang, W.-C., Chan, W.-L., Tsai, W.-T., Chen, G.-Z., Lee, C.-J., Chiu, C.-M., Chien, C.-H., Wu, M.-C., Huang, C.-Y., Tsou, A.-P., and Huang, H.-D. (January, 2011) miRTarBase: a database curates experimentally validated microRNA-target interactions.. *Nucleic acids research,* **39**(Database issue), D163–9.
5. Spizzo, R., Nicoloso, M. S., Croce, C. M., and Calin, G. A. (May, 2009) SnapShot: MicroRNAs in Cancer.. *Cell,* **137**(3), 586–586.e1.
6. Zaman, N., Li, L., Jaramillo, M. L., Sun, Z., Tibiche, C., Banville, M., Collins, C., Trifiro, M., Paliouras, M., Nantel, A., O'Connor-McCourt, M., and Wang, E. (October, 2013) Signaling Network Assessment of Mutations and Copy Number Variations PredictBreast Cancer Subtype-Specific Drug Targets. *CellReports,* **5**(1), 216–223.
